# Disruption of Nuclear‐Cytoskeletal Linkage by Coil‐1a LMNA Mutations in Emery–Dreifuss Muscular Dystrophy

**DOI:** 10.1002/jcsm.70234

**Published:** 2026-02-17

**Authors:** So‐mi Kang, Ran Kim, Tae‐Gyun Woo, Soyoung Park, Yeongseon Ji, Ha Eun Kim, Yu Jin Jeong, Jeongmo Kim, Yeonhee Kim, Woochul Chang, Bae‐Hoon Kim, Bum‐Joon Park

**Affiliations:** ^1^ Department of Molecular Biology Pusan National University Busan Korea; ^2^ Institute of Systems Biology, College of Natural Science Pusan National University Busan Korea; ^3^ Department of Biology Education Pusan National University Busan Korea; ^4^ Rare Disease R&D Center, PRG S&T Co. Ltd. Busan Korea

**Keywords:** EDMD, Emery‐Dreifuss muscular dystrophy, lamin A, muscular dystrophy, nucleus

## Abstract

**Background:**

Emery–Dreifuss muscular dystrophy (EDMD) is a progressive genetic myopathy that mainly affects the muscles used for movement (skeletal muscles) and the heart (cardiac muscles). The disease is frequently associated with mutations in genes encoding nuclear envelope proteins, most notably LMNA, which encodes lamin A—a critical structural component of the nuclear lamina. Lamin A plays a pivotal role in maintaining nuclear architecture and mechanotransduction. In contrast to most other cell types, nuclei in healthy skeletal muscle fibres are typically localized at the periphery of the myofiber. However, muscle biopsies from EDMD patients often reveal aberrant nuclear morphology and ectopic nuclear positioning, with nuclei clustered or mislocalized toward the centre of the myofiber. Despite these characteristic nuclear abnormalities, the molecular mechanisms underlying nuclear mispositioning in EDMD remain incompletely understood. In particular, the interaction networks between EDMD‐related mutant lamin A and other nuclear and cytoskeletal components that govern nuclear positioning are poorly characterized in the current literature.

**Methods:**

EDMD‐related lamin A variants (L35V, L38F or Y45C), which are located within the Coil‐1a domain, were overexpressed in RD cells. Mesenchymal stem cells (MSCs) were generated by redifferentiating induced pluripotent stem cells (iPSCs), which were derived from fibroblasts of an EDMD (L35P) patient. To investigate morphological and molecular abnormalities caused by mutations, immunofluorescence imaging, immunoblotting and subcellular fractionation were performed. Functional consequences of these morphological alterations were evaluated by assessing mechanotransduction signalling and cell viability.

**Results:**

EDMD‐related LMNA mutations (L35V, L38F, Y45C) in the Coil‐1a domain induced multilobular nuclear morphology, accompanied by a decrease in nuclear contour ratio (1.9–3.0‐fold vs. WT, *p* < 0.0001). Similarly, patient‐derived MSCs (L35P‐MSCs) exhibited a ~2.28‐fold decrease in contour ratio relative to healthy subject‐derived MSCs. Abnormal nuclear shape was associated with structural alterations in nuclear‐cytoskeletal proteins and nuclear positioning regulators. Mechanosensing activity, assessed by YAP1 nuclear translocation, was increased (~1.72‐fold vs. Nor‐CTRL, *p* < 0.01), and nuclear fragility under physical stress was elevated by ~20% (vs. Nor‐CTRL, *p* < 0.0001). Treatment with mutation‐specific ASOs in patient‐derived MSCs restored the contour ratio (~1.97‐fold vs. NC‐CTRL, *p* < 0.01), normalized nuclear‐cytoskeletal organization, reduced mechanosensing response (~1.65‐fold vs. NC‐CTRL, *p* < 0.01) and decreased nuclear fragility by ~11% (vs. NC‐CTRL, *p* < 0.0001).

**Conclusions:**

Our findings indicate that nuclear morphological alterations contribute to the impaired nuclear‐cytoskeletal integrity and nuclear positioning, which are closely linked to cellular mechanics and function. Mutation‐specific ASO treatment alleviated these nuclear defects, suggesting that ASO‐based therapeutic strategies may provide a mutation‐targeted approach to correcting nuclear abnormalities in EDMD.

## 1 Introduction

Lamins are intermediate filament proteins forming a mesh network beneath the inner nuclear membranes of metazoan cells [1]. Two general types of lamins, A‐type and B‐type, are essential for providing structural support to the nucleus and facilitating tethering with chromatin or other nuclear proteins [2, 3], S1. Lamin A is an A‐type lamin that arises from the *LMNA* gene. To date, a substantial number of lamin A mutations have been identified and reported in the literature. These mutations are known to cause laminopathies, including muscular dystrophies, dilated cardiomyopathy, lipodystrophies, neuropathies and premature aging syndromes. The most common group of laminopathies is muscular dystrophy, especially Emery–Dreifuss muscular dystrophy (EDMD) [2, 4], S2.

Multijoint contractures, slow progressive muscle wasting and cardiomyopathy with conduction defects characterize EDMD [5, 6], S3. EDMD has three patterns of inheritance: an X‐linked, autosomal dominant and, rarely, an autosomal recessive manner. In pioneering research, the first report on EDMD mentioned the X‐linked recessive form, which is linked to mutations in *EMD* encoding emerin [7]. The following research reported de novo autosomal dominant EDMD, which is caused by mutations in *LMNA*, encoding Lamin A/C [8, 9], S4. LMNA mutations that cause EDMD are found throughout the gene, most being point mutations resulting in amino acid substitutions. There is still no clear correlation between the site of mutation and the occurrence of EDMD.

Nuclear positioning is indispensable for normal intracellular organization and is controlled by nuclear envelope proteins and the cytoskeleton [10, 11, 12, 13], S5. Unlike other organs, nuclei in skeletal muscles are positioned at the periphery of the cell to protect them from contraction and maximize the distance between adjacent nuclei. This positioning is important for muscle function and maintenance [14, 15]. Mispositioned nuclei are associated with muscle dysfunction and disorders. BIN1 (amphiphysin‐2) is a conserved protein that plays a role in nuclear positioning by linking the nuclear envelope to the microtubule and actin cytoskeletons [16]. Impairment of BIN1 can alter nuclear shape and position in muscle cells. Mutations in BIN1 can cause myopathies, such as centronuclear myopathies (CNMs), characterized [17, 18, 19] by abnormal central positioning of nuclei in the skeletal muscle and myotonic dystrophy. EDMD patients are also known to possess misshapen nuclei prominently within the centre of the muscle fibre [15], S6. However, how the nuclei are mispositioned in muscle fibre without genetic mutations in nuclear positioning proteins, such as BIN1.

In this study, we investigated how LMNA variants in the Coil‐1A domain, which produce multilobular‐shaped nuclei, can aberrantly aggregate, disrupt the endogenous lamina and nuclear‐cytoskeleton system and alter BIN1 localization, ultimately leading to abnormal nuclear positioning. We further assessed the functional consequences of these morphological alterations by evaluating changes in gene expression, mechanotransduction and cellular viability. In parallel, we assessed the efficacy of a mutation‐specific antisense oligonucleotide (ASO) approach to correct irregular nuclear deformation in EDMD patient‐derived cells.

## 2 Methods

### 2.1 Cell Culture and Reagents

All methods performed in this study were approved by the Institutional Review Board (IRB) at Pusan National University (PNU) in accordance with relevant guidelines and regulations. Human rhabdomyosarcoma RD cells were obtained from the American Type Culture Collection (ATCC, Manassas, VA, USA) and maintained in Dulbecco's Modified Eagle's Medium (DMEM) supplemented with 10% foetal bovine serum (FBS) and 1% penicillin–streptomycin. Primary human fibroblast cells from EDMD patient (GM23780, 14‐year‐old male) and unaffected healthy control (GM00038, 9‐year‐old female) were obtained from Coriell Cell Repositories (Camden, NJ, USA) and maintained in Eagle's Minimal Essential Medium (EMEM) supplemented with 15% FBS and 2‐mM glutamine without antibiotics. All cell lines were incubated at 37°C with 5% CO_2_ and were utilized in our laboratory under study protocols approved by PNU IRB, in accordance with relevant guidelines and regulations.

### 2.2 Reprogramming Human Fibroblasts Into Induced Pluripotent Stem Cells (iPSCs)

iPSCs were established by reprogramming primary fibroblasts of an EDMD patient and an unaffected healthy individual using CytoTune‐iPS 2.0 Sendai Reprogramming Kit (#A16518, Life Technologies), which transiently expressed Oct4, Sox2, c‐Myc and Klf4. After approximately 3 to 4 weeks, iPSC colonies began to appear. These colonies were then manually picked and expanded on vitronectin‐coated 6‐well plates. Cells were maintained in a humidified incubator at 37°C with 5% CO_2_. The E8F culture medium was replaced every other day until the cells reached confluency.

### 2.3 Differentiating Human iPSCs Into Mesenchymal stem cells (MSCs)

To obtain MSCs from iPSCs, the STEMdiff Mesenchymal Progenitor kit (STEMCELL Technology, Canada) was used following the manufacturer's instructions. Firstly, iPSCs, derived from an EDMD patient and an unaffected healthy subject, were cultured in mTeSR1 medium. On Day 0, cells were ready for induction into early mesoderm progenitor cells by replacing TeSR medium with STEMdiff Mesenchymal Induction Medium. By Day 4, STEMdiff Mesenchymal Induction Medium was replaced with MesenCult‐ACF Medium to derive early MSCs. On Day 6, cells were passaged onto cultureware precoated with MesenCult‐ACF Attachment Substrate in MesenCult‐ACF Medium by Day 21.

### 2.4 Antibodies and Reagents

The antibody used for experiments included antibodies against Flag, GFP (1:3000; sc‐9996; Santa Cruz Biotechnology, USA), His, GST (1:5000; sc‐138; Santa Cruz Biotechnology, USA), lamin A/C (1:200 and 1:5000; 10298‐1‐AP; Proteintech, USA), phospholamin A/C‐S22 (1:2000; AP0777; ABclonal, USA), lamin B1 (1:100 and 1:2000; 12987‐1‐AP; Proteintech, USA), emerin (1:200 and 1:3000; sc‐25284; Santa Cruz Biotechnology, USA), vimentin (1:3000; AMF‐17b; DSHB, USA), BIN1 (1:1000; 14647‐1‐AP; Proteintech, USA), CLIP1 (1:100 and 1:1000; GTX117504; GeneTex, USA), Nesprin2 (1:100 and 1:500; GTX121928; GeneTex, USA), *α*‐tubulin (1:200 and 1:1000; 11 224‐1‐AP, 66031‐1‐Ig; Proteintech, USA), SUN1 (1:100 and 1:1000; HPA008461; Atlas Antibodies, Sweden), *γ*‐tubulin (1:100; T6557; Millipore, USA), YAP1 (1:200 and 1:1000; 66900‐1‐Ig; Proteintech, USA) and actin (1:10000; 66009‐1‐Ig; Proteintech, USA).

### 2.5 Purification of Recombinant Proteins

To obtain the Head‐Rod region of lamin A/C protein, *Escherichia coli* strain B834 (DE3; Novagen, USA) harbouring plasmids encoding lamin A/C fragment (residue 1–300) was cultured in M9 medium supplemented with L‐(+)‐selenomethionine. Protein expression was induced by 0.5‐mM isopropyl *β*‐d‐1‐thiogalactopyranoside (IPTG) at 30°C. Cells were harvested by centrifugation and resuspended in lysis buffer containing 20‐mM Tris–HCl (pH 8.0) and 150‐mM NaCl. Cells were disrupted with a sonicator. Then, debris was removed by centrifugation. The supernatant of lamin A/C fragment was loaded onto Ni‐NTA affinity agarose resin (Qiagen, The Netherlands), preincubated with lysis buffer. Target proteins were eluted with lysis buffer supplemented with 250‐mM imidazole. These eluted fractions were further purified using anion‐exchange chromatography (Hitrap Q HP, GE Healthcare, USA). A similar strategy generated the short N‐terminal region of lamin A/C (1–151), full‐length BIN1, and the N‐terminal region of SUN1 proteins. *E. coli* strain B21 (DE3) harbouring plasmids encoding lamin A/C fragment (1–151) was cultured in LB medium. Protein expression was induced by 0.5‐mM IPTG at 15°C. Cells were harvested by centrifugation and resuspended in lysis buffer containing 20‐mM Tris–HCl (pH 7.4). Cell debris was removed after disrupting the cells with the sonicator. Each fragment was loaded onto GSH‐agarose and then eluted using a buffer containing 10‐mM reduced glutathione after extensive washing.

### 2.6 Transfection of Vector Plasmids

Flag‐tagged wild‐type lamin A was obtained from Seoul National University, and EDMD‐related lamin A mutants (L35V, L38F, and Y45c) expression vectors were created by a single‐point mutation using flag‐tagged wild‐type lamin A. GFP‐tagged lamin A expression vector was provided by Misteli T. (National Cancer Institute, Frederick, MD, USA), and EDMD‐related lamin A mutant (L35P) was generated using GFP‐tagged lamin A. jetOPTIMUS (Polyplus Transfection, New York, USA) was used for the transfection of all vectors in RD cells. The expression vectors were mixed with jetOPTIMUS in a dedicated buffer. Following the replacement of the medium with fresh medium, the mixture, which had been incubated for 10–15 min, was added to the RD cells.

### 2.7 ASO Transfection

A L35P‐targeting (L35P‐ASO) 19‐mer ssRNA sequence (5′‐GGAGGACCCGCAGGAGCUC‐3′) and a nontargeting control (NC‐ASO) 19‐mer ssRNA sequence (5′‐GCACGAGCCAGAUGGGCAC‐3′) were synthesized by BIONEER (Rep. of Korea). The ASO was mixed with INTERFERin (Polyplus, France) transfection reagent according to the manufacturer's protocol. In this study, INTERFERin complexes at a 100‐ or 200‐nM ASO concentration were transfected in cells. The ASO dosages were determined according to established methods reported in the literature (S7). After transient transfection of vector plasmids (GFP‐tagged LA‐WT and LA‐L35P), RD cells were transfected with 100 or 200 nM of L35P‐ASO and NC‐ASO and 4 μL of INTERFERin in a 12‐well plate and incubated for 24 h. MSCs were transfected with 100 nM of a given ASO and 4 μL of INTERFERin in a 12‐well plate for 48 h.

### 2.8 Immunoblotting

Immunoblotting assays were designed under protocols approved by the PNU IRB in accordance with relevant guidelines. Radioimmunoprecipitation assay (RIPA) buffer (50‐mM Tris‐Cl, pH 7.5, 150‐mM NaCl, 1% NP‐40, 0.1% SDS and 10% sodium deoxycholate) was used for protein extraction from cells. After heat‐inactivation with sample buffer, proteins were subjected to sodium dodecyl sulphate‐polyacrylamide gel electrophoresis (SDS‐PAGE) and transferred to polyvinylidene difluoride (PVDF) membranes. Blotted membranes were blocked with 3% skimmed milk for 1 h and incubated for 2 h to overnight with specific primary antibodies, followed by incubation with horseradish peroxidase‐conjugated goat antimouse, goat antirabbit or mouse antigoat IgG secondary antibodies. Peroxidase activity was detected by chemiluminescence using an ECL kit (Advansta Inc., San Jose, CA, USA) following the manufacturer's instructions.

### 2.9 Pull‐Down Assays

His pull‐down and glutathione *S*‐transferase (GST) pull‐down assays were performed under protocols approved by the PNU IRB. To detect the interaction, the bead‐conjugated His‐tagged lamin A (1–300 or 1–151), bead‐conjugated GST‐tagged BIN1 (full length) and bead‐conjugated GST‐tagged SUN1 (N‐terminus) recombinant proteins were incubated with wild‐type lamin A and EDMD‐related lamin A mutants expressing RD cell lysates for 30 min at room temperature (RT). After washing three times with PBS buffer, the binding materials were collected and subjected to SDS‐PAGE and sestern blot assay.

### 2.10 Co‐Immunoprecipitation Assays

Whole‐cell lysates, which were cotransfected with V5‐tagged BIN1 and flag‐tagged lamin A variants, were incubated with anti‐V5 antibody at 4°C for 2 h, followed by incubation with protein A/G agarose beads at 4°C for 1 h. After centrifugation and washing with RIPA buffer, the immunocomplexes were separated by SDS‐PAGE and subjected to western blot assay. To analyse proteins interacting with lamin A/C in MSCs, a total of 500 μL of whole‐cell lysates were incubated with antilamin A/C antibody at 4°C overnight, followed by incubation with protein A/G agarose beads at 4°C for 1 h. After centrifugation and RIPA washing, the immunocomplexes were analysed by western blot analysis.

### 2.11 Immunofluorescence Assays

The immunofluorescence (IF) assays were performed under protocols approved by the PNU IRB, following relevant guidelines and regulations. For IF staining, cells were cultured on coverslips. Cells were washed with 1X PBS gently, fixed with cold 4% paraformaldehyde (PFA) for 1 h at 4°C, and permeabilized with 0.2% Triton X‐100 at RT for 10 min. After incubation with blocking solution (1% goat serum in 1X PBS) for 1 h, cells were incubated with antilamin A/C, antiflag, anti‐BIN1, anti‐CLIP1, anti‐Nesprin2, anti‐*α*‐tubulin, anti‐SUN1, anti‐*γ*‐tubulin, antiemerin, and antilamin B1 in blocking solution overnight at 4°C. Then, cells were incubated with fluorescein isothiocyanate and rhodamine‐conjugated secondary antibodies at 4°C for 2 h. Nuclei were stained with DAPI (4, 6‐diamidino‐2‐phenylindole) at RT for 10 min. Lastly, cells were washed with 1X PBS three times, and coverslips were mounted with mounting solution (H‐5501; Vector Laboratories, USA). Immunofluorescence images were detected using fluorescence microscopy (Zeiss Apotome, Germany).

### 2.12 Subcellular Fractionation

Subcellular proteome extraction was performed using a ProteoExtract kit (Millipore, USA) following the manufacturer's instructions. After removing the culture medium, cells were washed and overlayed with the mixture of Extraction Buffer 1 (EB1) and protease inhibitor cocktail (PI) for 10 min at 4°C with gentle agitation. Secondly, cells were overlayed with EB2 mixture for 30 min at 4°C with gentle agitation after transferring the supernatant of EB1 to a clean tube. Then, the supernatant of EB2 was transferred to a clean tube. The cells were overlayed with the mixture of EB3, including nuclease, and gently collected the remaining cellular material into a clean tube. After centrifugation for 10 min at 6000 g, 4°C, the supernatant of the mixture of EB3 was transferred to a clean tube. The pellet of cellular material was gently resuspended with the mixture of EB4. Then, the fractions were subjected to western blot assay.

### 2.13 Nucleic and Cytoplasmic Protein Extraction With NP‐40 Lysis Buffer

NP‐40 lysis buffer (50‐mM Tris‐Cl, pH 7.5, 150‐mM NaCl and 1% NP‐40) was used for protein extraction from cells. Cells were overlayed with NP‐40 lysis buffer and gently collected into a clean tube. After centrifugation for 5 min at 3000 g, 4°C, the supernatant was transferred to a clean tube. The pellet was resuspended with RIPA buffer with vortexing. The extracts were subjected to SDS‐PAGE and western blot assay.

### 2.14 MTT Assay

To assess cell viability, cells were incubated with 0.5‐mg/mL MTT solution (Calbiochem) at 37°C for 4 h. After removing the remaining solution, the resulting formazan crystals were dissolved in 200 μL of DMSO, and absorbance was measured at 540 nm.

### 2.15 Physical Simulation

Cells (0.4 × 10^4^) were seeded onto 12‐mm round cover glasses (thickness 0.13–0.17 mm). After cell attachment, the coverslips were inverted. To apply physical stress, the inverted coverslips were gently suctioned to remove the medium, allowing the cell layer to fully adhere to the bottom surface. A second coverslip was then placed on top of the cell‐seeded coverslip, and fresh medium was added, followed by incubation for 24 h.

### 2.16 Quantification of Contour Ratio

To quantify nuclear contour irregularity, the area and circumference of each nucleus were measured using ImageJ software. The nuclear contour ratio was then calculated using the formula 4 × π × (area)/(circumference) [2], which provides a numeric value reflecting the degree of nuclear shape distortion.

### 2.17 Mechanotransduction Assay

To assess YAP1 translocation under stress condition, cells were seeded onto cover glasses and allowed to fully attach. To induce mild oxidative stress, 10‐μM CoCl_2_ was added 2 h prior to fixation. Following treatment, cells were fixed and immunostained with an anti‐YAP1 antibody. The subcellular localization of YAP1 was then analysed using fluorescence microscopy.

### 2.18 Statistical Analysis

All experiments were performed in at least two independent biological replicates, and each experiment was conducted separately. All values are presented as mean ± standard deviation (SD), and the exact number of biological replicates and related information are provided in the corresponding figure legends. Statistical significance in this study was determined using an unpaired *t*‐test when two conditions needed to be compared. All the graphs were created using GraphPad Prism 10 programme. Figure 6 was illustrated with BioRender.

## 3 Results

### 3.1 EDMD‐Related Lamin a Variants Display Multilobular Shaped Nuclei and Impairment of Binding Affinity

**FIGURE 1:**
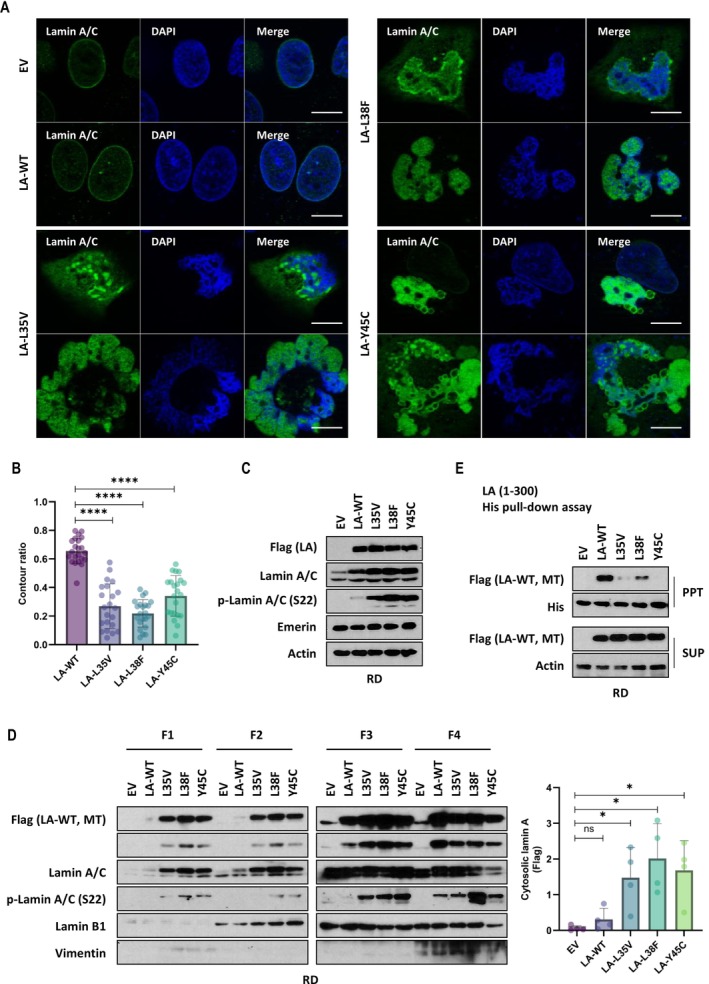
EDMD‐associated lamin A mutants show multilobular‐shaped nuclei. RD cells expressing wild‐type lamin A (LA‐WT) and EDMD‐related lamin A variants (LA‐L35V, LA‐L38F and LA‐Y45C) were fixed and immunostained for lamin A/C (green) and stained with DAPI (blue). Scale bar, 10 μm (A). The bar graph shows the ratio of nuclear contouring in wild‐type and mutant lamin A‐expressing RD cells. Cells were visualized 24 h after transfection, and the nuclear contouring ratio was quantified from photomicrographs using ImageJ (*n* = 22; each dot represents a mean value from a single fluorescence image slide acquired at x20 magnification, containing approximately 45–50 nuclei. *n* indicates the number of image slides analysed) ****p < 0.0001 by unpaired *t*‐test (B). RD cells were transiently transfected with an empty vector (EV), wild‐type lamin A or mutant lamin A vectors for western blot analysis. Whole‐cell lysates from EV, LA‐WT, LA‐L35V, LA‐L38F and LA‐Y45C expressing RD cells were subjected to sodium dodecyl sulphate‐polyacrylamide gel electrophoresis (SDS‐PAGE) and immunoblotting with antibodies targeting endogenous lamin A/C, phosphospecific lamin A/C (S22) and emerin. Actin served as a loading control (C). Western blot analysis of protein levels in cytoplasmic, membrane, nuclear and cytoskeletal fractions from RD cells expressing wild‐type lamin A or mutant lamin A. F1, cytoplasmic fraction; F2, membrane fraction; F3, nuclear fraction; F4, cytoskeletal fraction. Vimentin: a marker of F4 fraction in the fractionation assay. The bar graph (right) indicates the relative expression of cytosolic flag‐tagged lamin A in RD cells (*n* = 4) *p<0.05; ns: not significant by unpaired *t*‐test. Graph data were quantified using ‘Band Peak Quantification’ module in ImageJ software (D). His pull‐down assay using whole lysates of RD cells expressing flag‐tagged LA‐WT, LA‐L35V, LA‐L38F or LA‐Y45C. Each lysate was incubated with bead‐conjugated His‐tagged lamin A (1–300 region) recombinant proteins (E).

Fibroblasts derived from EDMD often possess aberrant and irregular nuclear architecture. In order to study the morphological expression of nuclear lamin in EDMD‐associated lamin A mutations, we decided to use RD cells as a primary model system in our investigation. RD is a rhabdomyosarcoma cell line of human skeletal muscle origin. Despite its tumorigenic nature, its muscle lineage and high transfection efficiency make it suitable for this study, and thus, it was used as a primary experimental cell line. To analyse particular EDMD‐related lamin A mutants, we generated three different flag‐tagged lamin A variant plasmids carrying EDMD‐associated point mutations. The mutations were L35V, L38F and Y45C, which are located in Coil 1a domain, and wild‐type lamin A constructs were used as controls. We performed transfection of plasmid DNA into RD cells and examined them 24 h after transient transfection. Significant changes in subcellular lamin A/C distribution were observed in the case of L35V, L38F and Y45C compared with empty‐control and wild‐type lamin A. Multilobular‐shaped nuclei and nuclear blebbing were accumulated in EDMD‐related mutants (Figure 1A and Figure S1A). Lamin A normally lines the inside of the nuclear membrane. However, it was observed that lamin A/C was diffused in the nucleoplasm and cytoplasm in EDMD‐related mutants (Figure 1A). To analyse irregular nuclear shapes, a contouring ratio is calculated by measuring nuclear circularity (Figure 1B and Figure S1B). There were no visible changes in the overall level of lamin A/C protein in the transfected cells. The expression of emerin, another nuclear membrane protein, was not also changed. But the level of phospholamin A/C (S22) protein was significantly increased in three mutants compared with wild‐type (Figure 1C and Figure S2A). Ser22‐phosphorylated lamin A/C is reported to localize in the nuclear interior and promote lamin A/C disassembly and degradation [20], S8, S9. Next, we assessed the subcellular fractionation to study the distribution of lamin A proteins by EDMD‐related mutation. The fraction revealed that EDMD‐related lamin A mutants easily spread out to cytoplasmic and membrane regions compared with wild‐type lamin A (Figure 1D). These results were confirmed by fractionation utilizing NP‐40 lysis buffer, a nonionic detergent that breaks open cell membranes to extract cytoplasmic proteins (Figure S2B,C). Because nuclear alterations have been reported to be associated with cell survival [21, 22, 23], we investigated whether EDMD‐related lamin A mutations impair cell survival. RD cells were subjected to long‐term (5 days) overexpression of wild‐type or mutant lamin A, and cell viability was assessed using MTT assay. Notably, EDMD‐related lamin A mutants markedly reduced cell viability compared with wild‐type lamin A (Figure S2D). To assess whether assembly of lamin A/C is changed in the mutated condition, we incubated purified recombinant His‐tagged lamin A fragment protein (aa 1–300 amino acids) with full‐length wild‐type lamin A or EDMD‐related lamin A mutants whole‐cell lysates. Immunoblotting showed that assembly of lamin A was lost in all lamin A mutants (Figure 1E and Figure S2E). However, each lamin A mutant, which lacks affinity to wild‐type lamin A, strongly bound to itself or other mutants (Figure S3).

To determine whether the effects observed in RD cells were reproducible in another muscle‐lineage system, we also examined mouse myoblast C2C12 cells by expressing EDMD‐related lamin A mutants. Similar to RD cells, C2C12 cells with lamin A mutations exhibited abnormal nuclear morphology, including nuclear lobulation (Figure S4A,B). In addition, in C2C12 cells, EDMD‐related lamin A mutants also failed to properly bind to wild‐type lamin A (Figure S4C).

Overall, the data showed that EDMD‐related lamin A mutants in the Coil‐1a domain caused abnormal self‐aggregation and mislocalization of lamin A, which subsequently led to the formation of multilobular shaped nuclei and ultimately resulted in reduced cell viability.

### 3.2 Multilobular Shaped Nuclei by Lamin a Mutation Induce Unusual Changes in BIN1, a Nuclear Positioning Protein

**FIGURE 2:**
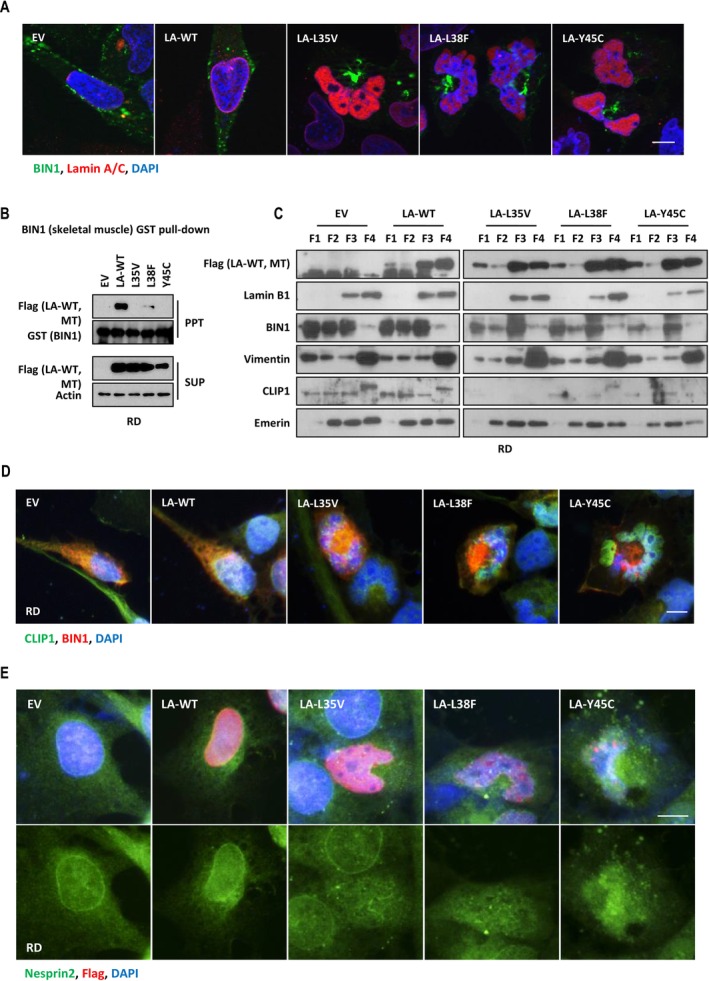
EDMD variants possessing multilobular‐shaped nuclei disintegrate BIN1, a nuclear positioning protein. Immunofluorescence (IF) assay for the analysis of BIN1 localization. Cotransfection of BIN1 with EV, LA‐WT, LA‐L35V, LA‐L38F or LA‐Y45C was performed in RD cells. RD cells were immunostained for lamin A/C (red) and BIN1 (green) and stained with DAPI (blue) after transfection (A). GST pull‐down assay using RD whole lysates expressing flag‐tagged LA‐WT, LA‐L35V, LA‐L38F and LA‐Y45C. Each lysate was incubated with bead‐conjugated GST‐tagged BIN1 (isoform 8, skeletal muscle) recombinant proteins (B). Western blot analysis of BIN1 protein in cytoplasmic, membrane, nuclear and cytoskeletal fractions from RD cells expressing wild‐type lamin A or mutant lamin A. F1, cytoplasmic fraction; F2, membrane fraction; F3, nuclear fraction; F4, cytoskeletal fraction (C). Immunofluorescence analysis of CLIP1 (green) and BIN1 (red) expression in transiently transfected RD cells with EV, LA‐WT, LA‐L35V, LA‐L38F and LA‐Y45C (D). Immunofluorescence analysis comparing distribution of endogenous Nesprin2 in RD cells expressing wild‐type or mutant lamin A. Transiently transfected RD cells were immunostained for flag (red) and Nesprin2 (green) and stained with DAPI (blue) (E). Scale bar, 10 μm.

Next, we investigate the molecular pathway linking irregularly shaped nuclei by lamin A mutations to abnormal nuclear positioning in EDMD. BIN1 (amphiphysin‐2) has an evolutionarily conserved role in nuclear positioning. Alteration in BIN1 is known to cause CNM characterized by abnormal nuclear positioning and shape [16]. We tested whether EDMD‐related lamin A mutants act to influence BIN1. Using RD cells transfected with plasmids encoding wild‐type lamin A and three EDMD‐related lamin A variants, we observed an obvious impact on BIN1 localization. BIN1 in lamin A mutants expressing cells was aggregated in the inside corner of the nuclei (Figure 2A). Also, the BIN1 protein failed to interact with lamin A‐L35V, L38F or Y45C compared with wild‐type lamin A (Figure 2B and Figure S5A–D). This binding deficit also occurred in C2C12, a mouse myoblast (Figure S5E). To investigate subcellular BIN1 localization in EDMD‐related lamin A mutants, a cellular fractionation was performed. The fraction revealed that cytoplasmic and membrane BIN1 were decreased in EDMD compared with empty‐control and wild‐type lamin A (Figure 2C and Figure S5F). BIN1 interacts with Nesprin2, a nuclear envelope protein and CLIP1, a microtubule plus‐end‐binding protein, to link the nuclear envelope to the actin and microtubule cytoskeleton, leading to orchestrating nuclear positioning and shape [16]. CLIP1 normally localizes to the cytoskeleton. However, the expression and the localization of CLIP1 are altered in EDMD‐related lamin A mutants (Figure 2C,D, and Figure S6). Nesprin2 was localized at the nuclear envelope in empty‐control and wild‐type lamin A, but it was destabilized in the lamin A‐L35V, L38F and Y45C (Figure 2E).

Collectively, these data suggest that multilobular shaped nuclei by lamin A mutants induce mislocalization of nuclear positioning proteins, leading to mispositioning of nuclei in EDMD.

### 3.3 The Interplay Between Nuclear Envelopes and Cytoskeletons Disintegrates in EDMD‐Related Lamin a Variants

**FIGURE 3:**
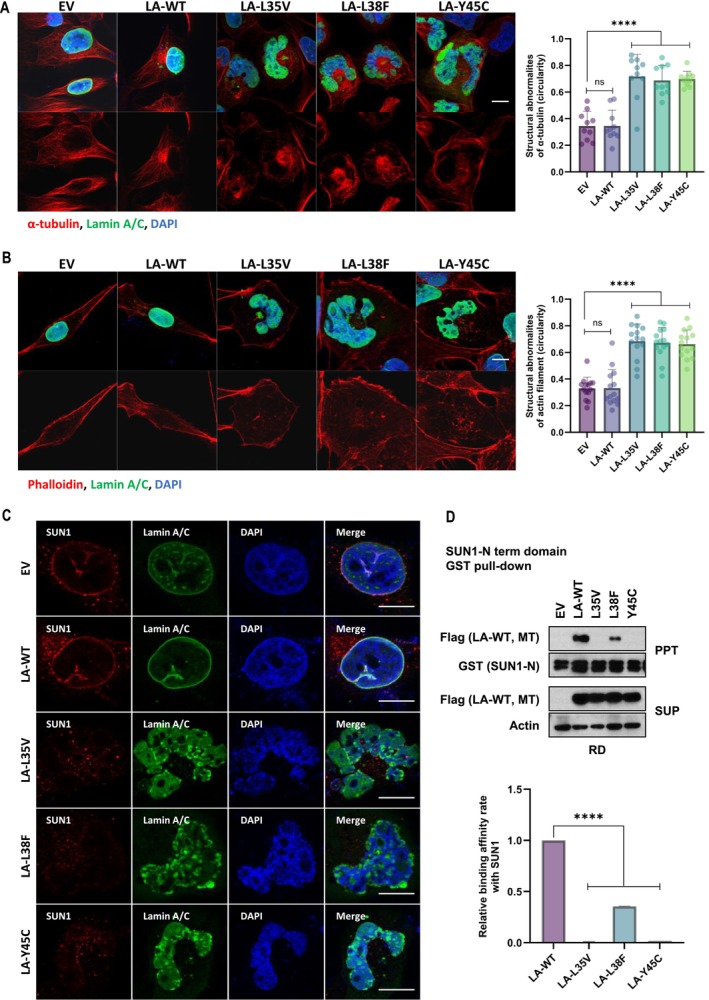
EDMD variants induce disruption of nuclear envelopes and cytoskeletons. Immunofluorescence (IF) analysis of microtubules (A) and actin filaments (B) in transiently transfected RD cells. RD cells were fixed and immunostained for lamin A/C (green) and *α*‐tubulin (red) (A). The actin filaments were stained with phalloidin (red) (B). The bar graphs show the relative structural abnormalities of *α*‐tubulin (A, right) or actin filament (B, right) in RD cells. The degree of structural abnormality was evaluated by ImageJ‐based circularity analysis of *α*‐tubulin and actin filaments (*n* = 14, each dot represents a mean value from a single fluorescence image slide acquired at x20 magnification, containing approximately 20–30 nuclei. *n* indicates the number of image slides analysed) ****p < 0.0001, ns: not significant by unpaired *t*‐test (A and B). Immunofluorescence analysis of SUN1, a nuclear envelope protein, in RD cells overexpressing EDMD variants. Transiently transfected RD cells were immunostained for SUN1 (red) and lamin A/C (green) (C). GST pull‐down assay using whole lysates of RD cells expressing flag‐tagged LA‐WT, LA‐L35V, LA‐L38F and LA‐Y45C. Each whole lysate was incubated with bead‐conjugated GST‐tagged SUN1 (N‐term region) recombinant proteins (D). The bar graph (lower) shows the relative binding affinity of SUN1 to lamin A mutants compared with wild‐type lamin A (*n* = 2, independent experiments) ****p < 0.0001, unpaired *t*‐test. Graph data were quantified using ‘Band Peak Quantification’ module in ImageJ software (D). The nuclei of all images were labelled with DAPI (blue). Scale bar, 10 μm.

The connection between the nuclear envelope and cytoskeleton is indispensable for nuclear positioning. Nesprins are located at the outer nuclear membrane and directly connect the nucleus to actin filaments [24], S10. CLIP1 regulates dynamics, growth, and bundling of the microtubule cytoskeleton [25, 26]. We also explored the cytoskeletal organization in RD cells because Nesprin2 and CLIP1 were impaired by EDMD‐related lamin A mutations (Figure 2D,E). To distinguish the effects of multilobular shaped nuclei by EDMD‐related lamin A mutations, we performed immunofluorescence staining to examine whether the two important components of the cytoskeleton, microtubules and actin filaments, were involved in morphological change. The immunofluorescence images of *α*‐tubulin (red) revealed the architectural alterations of microtubules in lamin A‐L35V, L38F and Y45C‐expressed cells compared with empty‐control and wild‐type lamin A (Figure 3A). *γ*‐tubulin, a protein that helps the assembly of microtubules, was also stuck in the inside corner of the nuclei, accompanied by BIN1 (Figure S7). On the other hand, the actin filaments exhibited a dense meshwork of altered polarization in EDMD‐related lamin A mutant‐expressed cells (Figure 3B).

SUN1 protein in the inner nuclear membrane interacts with nuclear lamin A to provide a connection between the nuclear lamina to the cytoskeleton [27, 28], S11. This connection is important for nuclear positioning and anchoring. Thus, we examined the correlation between SUN1 and EDMD‐related lamin A mutants. The fluorescence analysis showed that SUN1 is properly located on the inner nuclear membrane in empty‐control and wild‐type lamin A. However, in EDMD‐related lamin A mutants forming multilobular shaped nuclei, SUN1 structure was destroyed as well as the teardown of nuclear peripheral lamin A/C (Figure 3C). The N‐terminal domain of SUN1 binds to lamin A [28]. The interaction between the N‐terminal domain of SUN1 and wild‐type lamin A was confirmed by GST pull‐down assays. The interaction was weaker with EDMD‐related lamin A mutants than with wild‐type lamin A (Figure 3D). The expression of SUN1 protein was also degraded in lamin A mutants expressing RD cells (Figure S8).

These results indicate that multilobular shaped nuclei by EDMD‐related lamin A mutation can disrupt the nuclear cytoskeleton connection by destabilization of nuclear membrane proteins and cytoskeleton complex. Furthermore, these abnormalities can be linked to interaction failure with nuclear positioning proteins, such as the BIN1 protein, in EDMD‐related lamin A mutants.

### 3.4 EDMD Patient‐Derived MSCs Show Nuclear Defects and Nuclear Cytoskeleton Alterations

**FIGURE 4:**
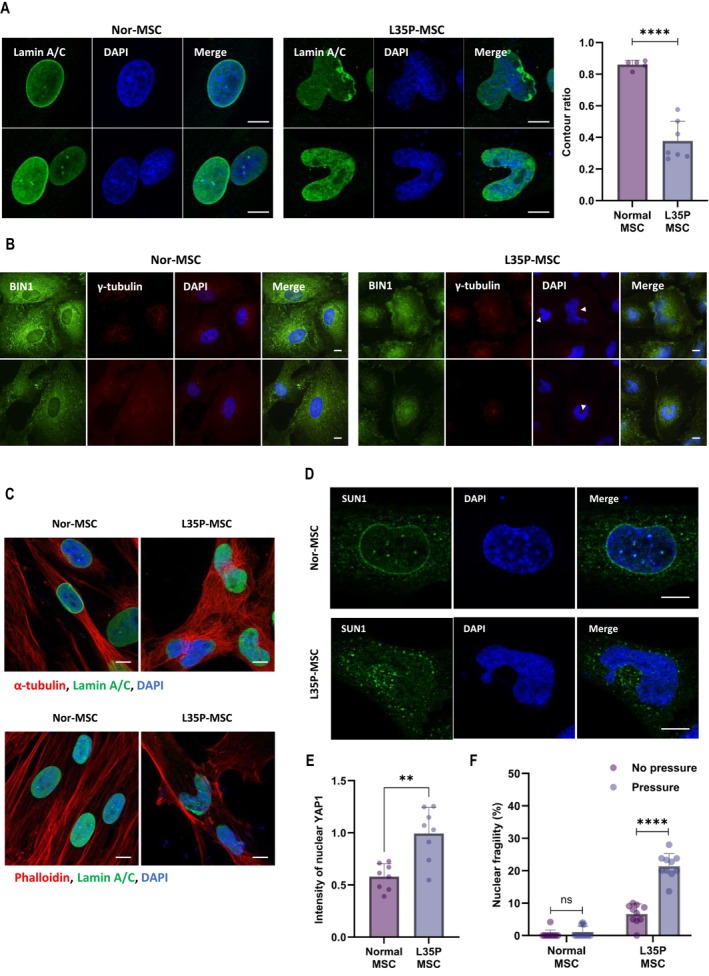
EDMD patient‐derived MSCs show multilobular shaped nuclei and impairment of nuclear cytoskeleton. Immunofluorescence (IF) analysis for nuclear morphology and lamin A/C (green) expression in EDMD patient‐derived mesenchymal stem cells (L35P‐MSCs) compared with healthy subject‐derived MSCs (Nor‐MSCs). The nucleus was labelled with DAPI (blue) (A). The bar graph (right) shows the ratio of nuclear contouring in Nor‐MSCs and L35P‐MSCs. The nuclear contouring ratio was quantified from photomicrographs using ImageJ (*n* = 5 (Normal), *n* = 7 (L35P), each dot represents a mean value from a single fluorescence image slide acquired at x20 magnification, containing approximately 35–40 (Normal) or 25–30 (L35P) nuclei. *n* indicates the number of image slides analysed) ****p < 0.0001 by unpaired *t*‐test (A). Immunofluorescence analysis for BIN1 (green) and *γ*‐tubulin (red) expression in L35P‐MSCs compared with healthy subject‐derived MSCs. The white arrowhead indicates *γ*‐tubulin (B). Immunofluorescence analysis of *α*‐tubulin (upper) and actin filaments (lower) in Nor‐MSCs and L35P‐MSCs. Cells were fixed and immunostained for lamin A/C (green) and *α*‐tubulin (red, upper). The actin filaments were stained with phalloidin (red, lower) (C). Immunofluorescence analysis of SUN1 expression in Nor‐MSCs and L35P‐MSCs. Cells were immunostained with SUN1 (green) (D). The bar graph indicates the intensity of nuclear YAP1 in Nor‐MSCs and L35P‐MSCs under stress conditions (CoCl_2_ 10 μM for 2 h). The intensity of nuclear YAP1 was quantified from photomicrographs using ImageJ (*n* = 8, each dot represents a mean value from a single fluorescence image slide acquired at x20 magnification, containing approximately 20–30 nuclei. *n* indicates the number of image slides analysed) **p < 0.01 by unpaired *t*‐test (E). The graph shows the percentage of nuclear fragility, with or without pressure, in Nor‐MSCs and L35P‐MSCs. Nuclear fragility was quantified by counting the number of nuclei exhibiting nuclear blebbing or rupture under physical stimulation (*n* = 10, each dot represents a mean value from a single fluorescence image slide acquired at x20 magnification, containing approximately 20–30 nuclei. *n* indicates the number of image slides analysed) ****p < 0.0001, ns: not significant by unpaired *t*‐test (F). The nuclei of all images were labelled with DAPI (blue). Scale bar, 10 μm.

Next, to confirm our aim of this study in EDMD patient‐derived cells, we analysed fibroblasts obtained from a clinically affected person who is heterozygous for a de novo T > C transition at nucleotide 104 in exon 1 of the LMNA gene (104 T > C), resulting in the substitution of proline for leucine at codon 35 (Leu35Pro (L35P)). Fibroblast from a healthy individual was also used for control. However, there was a mild morphological alteration between fibroblasts from the EDMD patient and the healthy subject. The nuclear size of EDMD patient‐derived fibroblasts was larger than healthy subject‐derived fibroblasts (Figure S9A). Then, we generated induced pluripotent stem cells (iPSCs) from fibroblasts through reprogramming. Fibroblast‐derived iPSCs were differentiated into MSCs, which have the potential to differentiate into skeletal muscle cells (Figure S9B–D). Interestingly, the MSCs (L35P‐MSCs) derived from the EDMD patient show multilobular shaped nuclei like RD cells transiently transfected with plasmids encoding lamin A‐L35V, L38F and Y45C (Figures 1A and 4A). Also, BIN1 and *γ*‐tubulin proteins were stuck in the inside corner of the nuclei of L35P‐MSCs (Figure 4B). The immunofluorescence images of microtubules and actin filaments revealed the architectural alterations of the cytoskeleton in L35P‐MSCs compared with MSCs (Nor‐MSCs) derived from a healthy normal subject (Figure 4C and Figure S10A,B). Also, multilobular‐shaped nuclei in L35P‐MSCs showed the structural disruption of nuclear membrane proteins. The SUN1 protein was fractured in L35P‐MSCs (Figure 4D). Also, emerin and lamin B1, the nuclear envelope proteins helping maintain the nuclear architecture and mechanics, were still localized to the nuclear envelope but, in some patient‐derived cells, were absent from the nucleus (Figure S11A,B).

To explore whether the effects of lamin A mutations extend beyond nuclear morphology to the expression of proteins involved in nuclear‐cytoskeletal architecture and nuclear positioning, we analysed both MSCs by western blot. Endogenous lamin A/C level was relatively reduced in L35P‐MSCs compared with Nor‐MSCs. Consistently, the expression of SUN1 and CLIP1 was also decreased, whereas lamin B1, Nesprin2 and BIN1 showed no appreciable differences between the two groups. In addition, L35P‐MSCs exhibited substantially lower levels of H3K9ac than Nor‐MSCs (Figure S12A). H3K9ac is a histone modification associated with transcriptionally active chromatin and is involved in key cellular processes such as gene regulation, transcription, cell cycle progression and differentiation [29], S12. The abnormal reduction of H3K9ac in L35P‐MSCs suggests impaired chromatin regulation under mutant lamin A conditions.

Moreover, as observed in RD cells, L35P‐MSCs also showed reduced protein interactions. Specifically, immunoprecipitation assays demonstrated that the binding of lamin A/C to SUN1 or to BIN1 was decreased in L35P‐MSCs compared with Nor‐MSCs (Figure S12B,C).

Nuclear and cytoskeletal abnormalities can markedly influence cellular function and mechanics, including mechanotransduction signalling and nuclear stiffness [30, 31], S13, S14. Thus, we next aimed to investigate whether the morphological changes caused by EDMD‐related lamin A mutations disrupt cellular mechanics or function in MSCs. Under stress conditions, including oxidative stress, YAP nuclear localization can be induced [32, 33], S15. To investigate whether mild oxidative stress more prominently affects YAP1 nuclear translocation in L35P‐MSCs compared with Nor‐MSCs, we exposed both MSCs to CoCl_2_ for 2 h and examined YAP1 localization using immunofluorescence analysis. Compared with Nor‐MSCs, L35P‐MSCs exhibited a markedly higher level of nuclear YAP1 under mild oxidative stress, indicating that EDMD‐associated lamin A mutations enhance mechanosensitive responses to mild stress conditions (Figure 4E and Figure S13A). Furthermore, to assess whether morphological alterations induced by lamin A mutations also affect nuclear stiffness, we applied identical physical pressure to both Nor‐MSCs and L35P‐MSCs and measured nuclear fragility. Nuclear fragility reflects the reduced mechanical stability of the nucleus, leading to deformation such as nuclear blebbing or nuclear rupture [30, 34, S16]. Under basal conditions, L35P‐MSCs exhibited higher nuclear fragility than Nor‐MSCs. When physical pressure was applied, nuclear fragility was further exacerbated in L35P‐MSCs, indicating a significantly reduced nuclear envelope stability compared with normal controls (Figure 4F and Figure S13B).

These results indicate that multilobular shaped nuclei by EDMD‐related lamin A mutations can disrupt the nuclear cytoskeleton by impairing nuclear envelope proteins and nucleocytoskeletal connections, which in turn affect mechanical properties and cellular function.

### 3.5 A Selective ASO Targeting a Single‐Point Mutation of LMNA Improves the Characteristics of EDMD Cells

**FIGURE 5:**
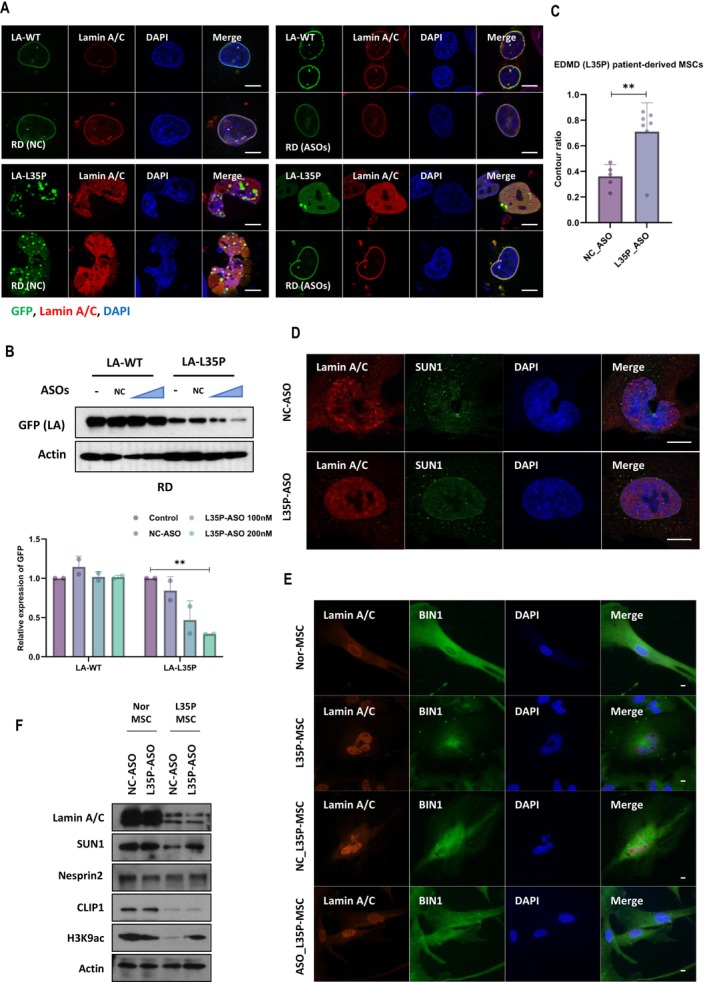
ASOs targeting LMNA‐L35P correct nuclear abnormalities. Immunofluorescence (IF) analysis of nuclear morphology and distribution of lamin A after treatment with ASOs. RD cells expressing GFP‐tagged LA‐WT or LA‐L35P were posttransfected with negative control ASO (NC‐ASO) and specific ASO targeting LMNA‐L35P (L35P‐ASO) for 24 h and immunostained for lamin A/C (red) and stained with DAPI (blue) (A). Western blot analysis of GFP‐tagged wild‐type lamin A and L35P‐lamin A expression after ASO treatment. RD cells were transfected with vectors expressing GFP‐lamin A (WT or L35P), followed 24 h later by treatment with either NC‐ASO or L35P‐ASO at concentrations of 100 or 200 nM. Whole‐cell lysates from ASOs‐treated LA‐WT or LA‐L35P expressing RD cells were subjected to SDS‐PAGE and immunoblotting with antibodies targeting GFP (LA‐WT or LA‐L35P) and actin. The graph (below) shows the relative GFP expression after ASO treatment. ‘Control’ means nontreated (*n* = 2, independent experiments) **p < 0.01 by unpaired *t*‐test (B). The bar graph shows the ratio of nuclear contouring after ASO treatment in L35P‐MSCs (*n* = 12, each dot represents a mean value from a single fluorescence image slide acquired at x20 magnification, containing approximately 25–30 nuclei. *n* indicates the number of image slides analysed) **p < 0.01 by unpaired *t*‐test (C). Immunofluorescence analysis for nuclear morphology after ASO treatment in L35P‐MSCs. Cells were immunostained for lamin A/C (red) and SUN1 (green) after treatment with NC‐ASOs and L35P‐targeting ASOs for 48 h (D). Immunofluorescence analysis for BIN1 expression after ASO treatment (48 h) in L35P‐MSCs. Cells were immunostained for lamin A/C (red) and BIN1 (green) and stained with DAPI (blue) for nuclei (E). Expression of SUN1, Nesprin2, CLIP1 and H3K9ac was examined by western blot in Nor‐MSCs and L35P‐MSCs following 48 h of ASO treatment (100 nM). The expression of SUN1, Nesprin2 and H3K9ac was increased after LMNA‐L35P targeting ASO in L35P‐MSCs (F). Scale bar, 10 μm.

Then, we examined whether morphological normalization could be restored by treatment with a mutant‐specific ASO. To target L35P‐LMNA specifically, we designed a 19‐base‐long ASO (L35P‐ASO) targeting cytosine at nucleotide 104 in exon 1 of the LMNA gene. We first tested the specificity of L35P‐ASO in RD cells transiently transfected with GFP‐tagged plasmids encoding wild‐type lamin A or L35P‐lamin A. Remarkably, L35P‐ASO effectively reduced L35P‐lamin A expression without affecting wild‐type lamin A expression (Figure 5A,B). L35P‐ASO treatment alleviated the reduction in cell viability that was caused by long‐term overexpression of the mutant lamin A in RD cells (Figure S14A). The multilobular shaped nuclei by LMNA‐L35P mutation were normalized by L35P‐ASO treatment (Figure 5A). Then, we also examined the recovery effects of L35P‐ASO in EDMD patient‐derived MSCs. The L35P‐ASO normalized multilobular shaped nuclei into round or oval shaped ones in L35P‐MSCs (Figure 5C,D, and Figure S14B). The SUN1 also settled back to its normal position after ASO treatment (Figure 5D). Accompanied by structural normalization of nuclei, BIN1 localization was recovered by ASO‐treated L35P‐MSCs, similar to healthy normal MSCs (Figure 5E). ASO treatment also restored the reduced protein levels of SUN1 and H3K9ac in L35P‐MSCs (Figure 5F and Figure S14C). Although L35P‐ASO did not alter the total protein levels of Nesprin2 or BIN1, it could restore their binding to lamin A/C in L35P‐MSCs (Figure 5F and Figure S14D,E). In addition, ASO treatment alleviated cellular defects observed in L35P‐MSCs, including cytoskeletal disorganization (Figure S15A,B), enhanced mechanosensitive responses to oxidative stress (Figure S16A,B) and increased nuclear fragility (Supporting Information: S17A,B).

These results demonstrate that ASO treatment can be effective in L35P‐MSCs in reducing L35P‐lamin A specifically and ameliorating altered morphology and impaired cellular function.

## 4 Discussion

In this study, we discovered that particular EDMD‐related lamin A mutants formed multilobular‐shaped nuclei, which in turn impaired nuclear‐cytoskeleton architecture and mislocalization of nuclear positioning proteins. These structural abnormalities were accompanied by impaired cellular mechanics and functional alterations, suggesting that defects in nuclear morphology extend beyond architectural disorganization to functional consequences at the cellular level. These results are notably consistent with pathological phenotypes observed in muscle biopsies from EDMD patients, which frequently exhibit misshapen and mispositioned nuclei, accompanied by muscle damage and weakness.

EDMD is a rare genetic disease that affects nuclear structure and positioning and is caused by nuclear envelope genes, including EMD, LMNA, FHL1, SUN1, SUN2 and TTN [35], S17, S18. Especially, a high proportion of disease‐linked point mutations in LMNA have been identified in EDMD [5, 35]. There is a high degree of variability in the clinical symptoms and age of onset. However, most of the point mutations in LMNA have a lack of case reports and data. Thus, we first characterized the nuclear morphologies of several EDMD‐related lamin A mutants and figured out that lamin A‐L35V, L38F and Y45C, located in the Coil‐1a domain, cause multilobular‐shaped nuclei. Not all of the EDMD‐related lamin A mutations reveal severely deformed nuclei. Some have normal‐shaped nuclei like wild‐type lamin A, and others have mildly abnormal‐shaped nuclei in cells (Figure S18A). The location of a domain where the mutation occurs is not correlated with cellular phenotypes. We observed that multilobular‐shaped nuclei were highly connected to binding loss to wild‐type lamin A. The lamin A rod domain mutant R386K also caused multilobular‐shaped nuclei and loss of binding affinity to wild‐type lamin A. On the other hand, H222Y and L530P mutations in lamin A revealed normal‐shaped or mildly abnormal‐shaped nuclei and maintained interaction with wild‐type lamin A (Figure S18B,C). While causality has not been established, existing clinical reports imply that these results could correlate with the severity of pathological manifestations in EDMD patients. One case report announced that an EDMD patient with the L35P mutation in LMNA has muscle weakness and atrophy, which begins in infancy [S19]. The patients harbouring L35V and Y45C mutations also presented severe muscular dystrophy with onset in childhood [S20, S21]. However, another reported a middle‐aged EDMD patient with a homozygous 664C → T transition in the LMNA gene, resulting in a H222Y amino acid substitution. Both parents were carriers of the mutation but remained unaffected themselves [S22]. Although information on each mutation remains limited, available case reports cautiously suggest a potential association between the extent of nuclear deformation and the severity of clinical symptoms in EDMD patients.

**FIGURE 6:**
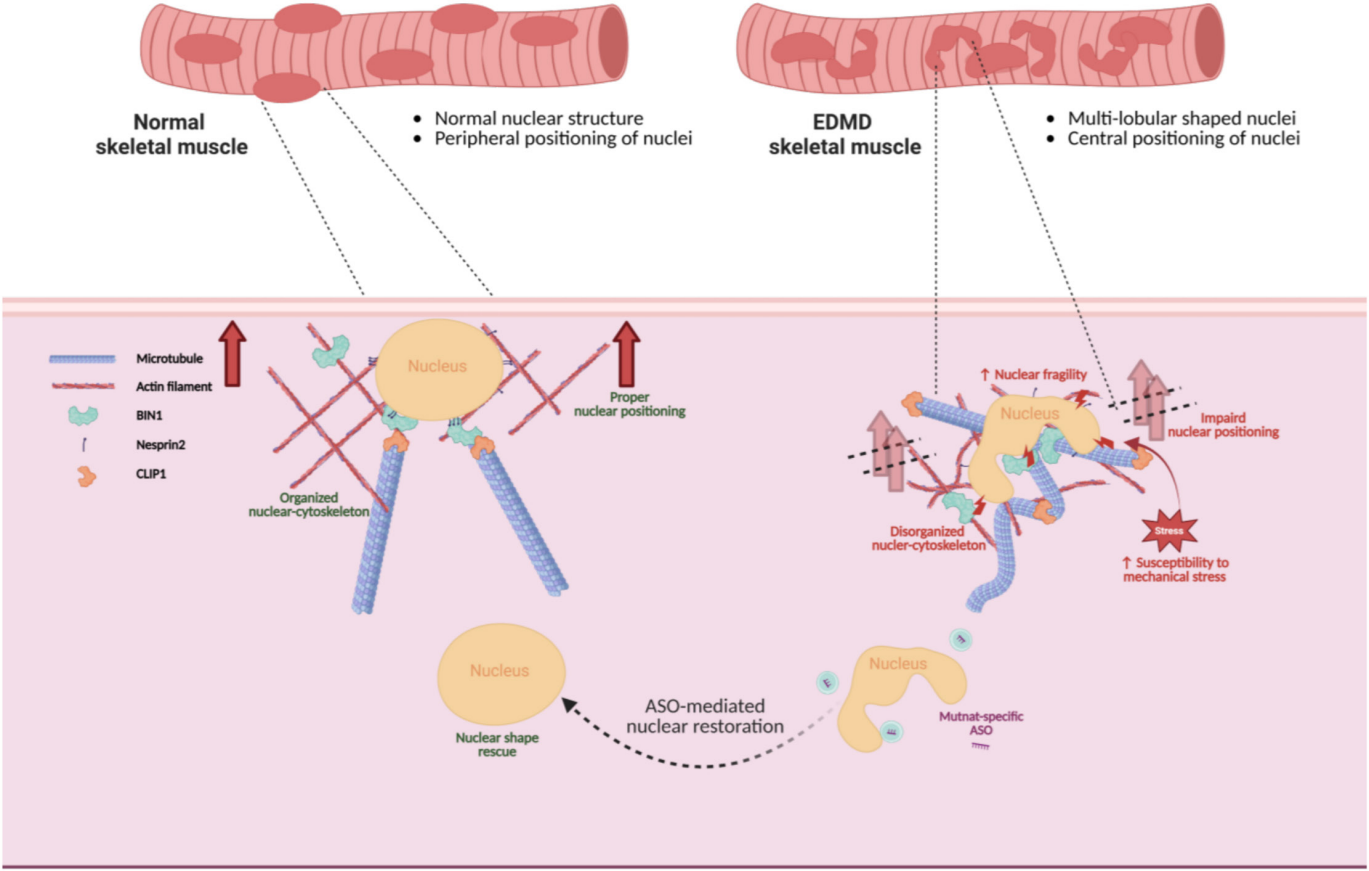
A schematic illustration of the aberrant processing mechanism in EDMD. Irregular nuclear deformation caused by EDMD‐related lamin A mutations leads to disruption of nuclear‐cytoskeletal organization, increased nuclear fragility and elevated susceptibility to mechanical stress. Mutation‐specific ASO treatment restores nuclear morphology and envelope integrity, alleviating EDMD‐associated defects.

The positioning of the nucleus within a cell is essential for maintaining cellular architecture, facilitating proper gene expression and ensuring effective cellular function. In skeletal muscle, nuclei are normally situated at the periphery of muscle fibres [36, 37]. This arrangement helps optimize contractile efficiency and protects the nuclei from the mechanical forces exerted during muscle contraction. Nuclear mispositioning is a pathological marker of EDMD. The BIN1 (Bridging Integrator 1) protein, also known as amphiphysin‐2, plays a significant role in nuclear positioning in muscle. BIN1 is known to interact with cytoskeletal components such as actin and microtubules. By connecting the nucleus to the cytoskeleton, BIN1 helps anchor the nucleus in the correct position within the muscle cells [16]. Abnormalities in BIN1 expression or function can result in mislocalization of the nucleus, contributing to pathological conditions. For example, mutations in the gene BIN1 (e.g., missense mutations in the BAR domain including K35N, D151N and R154Q and truncated mutations in the SH3 domain including Q434X and K436X) can cause abnormal positioning of nuclei in muscle fibres, a characteristic of CNM [17], S23. We hypothesized that multilobular‐shaped nuclei caused by LMNA mutations disrupt BIN1 protein, which might be associated with mispositioning of nuclei observed in muscle biopsies from patients with EDMD. In mutant cells, BIN1 was aggregated in the nuclear corners and did not effectively interact with the lamin A variants. These observations raise the possibility that nuclear shape abnormalities driven by LMNA mutations may indirectly affect the function of nuclear positioning machinery, leading to mispositioned nuclei in muscle cells without BIN1 mutations. Microtubule‐organizing centres (MTOCs) during muscle development shift from the centrosome to the nuclear envelope. This shift is crucial for establishing an organized microtubule network for proper nuclear positioning and muscle cell differentiation [38], S24. In cells, the MTOC utilizes *γ*‐tubulin to initiate microtubule growth [39], S25. Specifically, the *γ*‐tubulin ring complex (*γ*‐TuRC) is a key component of MTOCs [40], S26. This key component of MTOCs was also aberrantly stuck in the nuclear corners in EDMD mutant cells (Figure 4B). In parallel, the structure of microtubules and actin filaments was disorganized, and components of the LINC (linker of nucleoskeleton and cytoskeleton) complex, including Nesprin2 and SUN1, were disrupted in LMNA mutant cells. Notably, SUN1 not only exhibited impaired localization but also showed a reduction in protein expression in the presence of EDMD‐related lamin A mutations. Furthermore, the diminished interaction between lamin A and proteins associated with nuclear‐cytoskeletal coupling and nuclear positioning in EDMD patient‐derived MSCs is consistent with the aberrant cellular morphologies. Collectively, these findings imply that LMNA mutation‐induced multilobular‐shaped nuclei disrupt the integrity of nuclear‐cytoskeletal interactions, ultimately leading to defects in myonuclear positioning (Figure 6).

Morphological alterations of the nucleus are closely linked to cellular mechanics and function, including nuclear stiffness, mechanosensing, gene expression and cell viability [30, 31], S12. In line with this concept, the formation of multilobular shaped nuclei caused by EDMD‐related lamin A mutations is associated with reduced cell survival, decreased nuclear stiffness and aberrant mechanotransduction and gene expression. These findings suggest that structural deformation of the nucleus is not merely a morphological abnormality but has functional consequences that may contribute to disease pathology.

However, this phenomenon does not appear to be a universal consequence of all LMNA mutations. In cases where the nuclear envelope remained relatively intact or where multilobular nuclear morphology was not observed, such defects were absent. The Supporting Information data showing distinct phenotypes in other LMNA mutations, such as H222Y and L530P, suggest that the mechanism proposed in our study may not be broadly generalizable to all LMNA variants. A limitation of our study is that these findings may be restricted to specific LMNA mutations.

In this study, we utilized patient‐derived MSCs because they originate from the mesodermal lineage and possess the potential to differentiate into skeletal muscle cells. Thus, MSCs serve as a developmentally relevant intermediate model that allows investigation of early mechanonuclear defects prior to full myogenic differentiation, while providing experimental reproducibility. Nevertheless, we acknowledge that MSCs do not fully recapitulate the terminal muscle phenotype observed in EDMD. Therefore, we are currently extending our work by differentiating patient‐derived MSCs into myogenic cells to validate whether the morphological abnormalities and functional defects are similarly present in a more disease‐relevant muscle context.

To explore a therapeutic approach for LMNA‐related EDMD, we designed ASOs tailored to mutation‐specific LMNA sequence in order to correct mutation‐induced nuclear abnormalities. We made an L35P‐targeting ASO, which did not influence wild‐type lamin A (Figure 5B). The mutation targeting ASO effectively rescued the multilobular‐shaped nuclei and relocated nuclear envelope components and BIN1 protein in EDMD patient‐derived MSCs. In addition, ASO treatment reinstated defective protein interactions, promoted cytoskeletal reorganization and let to recovery of cellular functions, including mechanosensing and cell viability. Although this study demonstrates that ASO effectively reduces the expression of the L35P‐lamin A, suggesting its potential as a therapeutic strategy for EDMD, further validation is required. In particular, the ability of ASOs to specifically target single‐point mutations should be evaluated in muscle cell models and in LMNA‐related EDMD in vivo systems to confirm their therapeutic efficacy. In addition, careful consideration must be given to potential off‐target effects and adverse side effects. Since this study evaluated the therapeutic potential of ASO treatment in the context of a single LMNA mutation (L35P). Therefore, it remains to be determined whether this approach can be broadly applied to other LMNA mutations associated with EDMD. Future studies will be required to validate its broader applicability. Nevertheless, the present findings provide promising evidence that ASO‐mediated suppression of the mutant allele could serve as a viable therapeutic approach for EDMD. Although its efficacy may vary depending on the specific mutation, these findings raise the possibility that ASO offers a promising therapeutic avenue for EDMD associated with point mutations in the LMNA gene.

## Conflicts of Interest

Soyoung Park, Tae‐Gyun Woo, Bae‐Hoon Kim, and Bum‐Joon Park are employees of PRG S&Tech Co., Ltd. The remaining authors declare no conflicts of interest.

## Supporting information


**Data S1:** Supporting information.

**Data S2:** Supporting information.

**Figure S1:** Expression of EDMD‐associated lamin A mutants results in nuclei with a characteristic multilobulated morphology. RD cells expressing wild‐type (LA‐WT) and EDMD‐related lamin A mutants (LA‐L35V, LA‐L38F and Y45C) were immunostained for flag (green) and lamin A/C (red), and stained with DAPI (blue). Cells were visualized at 24 h after transfection. Scale bar, 10 μm (A). Schematic illustration showing the relationship between nuclear morphology and nuclear contour ratio. A value closer to 1 represents a rounded nucleus, whereas a lower ratio indicates a deformed nuclear shape (B).
**Figure S2:** EDMD‐associated lamin A mutants induce different spatial distributions. The bar graph indicates the relative expression of phospholamin A/C (S22) of wild‐type lamin A and EDMD‐related mutants in RD cells (*n* = 2, independent experiments) **p < 0.01 and *p < 0.05, ns: not significant by unpaired *t*‐test. Graph data were quantified using ‘Band Peak Quantification’ module in ImageJ software (A). Western blot analysis of nuclear and cytoplasmic fractions using NP‐40 lysis buffer. PEL, nuclear fraction; SUP, cytoplasmic fraction. Samples were centrifuged at 3000 rpm and the pellet (PEL) and supernatant (SUP) were collected separately (B). The stacked bar graph represents relative proportions of nuclear (dark purple) and cytosolic (light purple) fractions in wild‐type lamin A and EDMD‐related mutants. The stacked bar graph presents the quantification of relative Flag expression in the ‘PEL’ and ‘SUP’ lanes based on the western blot results shown in Figure S1D (C). The bar graph shows the relative cell viability after long‐term overexpression (5 days) in RD cells. Transfection was performed twice over a period of five days. Cell viability was measured by MTT assay (*n* = 2, independent experiments) ****p < 0.0001 and **p < 0.01 by unpaired *t*‐test (D). The bar graph shows the relative binding affinity of EDMD‐related mutants to lamin A protein compared with wild‐type lamin A (*n* = 2, independent experiments) **p < 0.01 and *p < 0.05 by unpaired *t*‐test. Graph data were quantified using ‘Band Peak Quantification’ module in ImageJ software (E).
**Figure S3:** EDMD‐associated lamin A mutants induce binding abnormalities. His pull‐down assay using bead‐conjugated His‐tagged lamin A‐L35V (A), lamin A‐L38F (B), or lamin A‐Y45C (C) recombinant proteins (1–151 region) with RD whole‐cell lysates expressing flag‐tagged LA‐WT, LA‐L35V, L38F and LA‐Y45C. Each bar graph (right) indicates the relative binding affinity to each lamin A‐mutant protein (*n* = 2, independent experiments) ***p < 0.001, **p < 0.01 and *p < 0.05 by unpaired *t*‐test. Graph data were quantified using ‘Band Peak Quantification’ module in ImageJ software.
**Figure S4:** EDMD‐associated lamin A mutants induce nuclear abnormalities and binding deficits in mouse myoblasts. C2C12 cells expressing flag‐tagged wild‐type lamin A (LA‐WT) and EDMD‐related mutants (LA‐L35V, LA‐L38F and LA‐Y45C) were fixed and immunostained for flag (green), lamin A/C (red) and stained with DAPI (blue). Scale bar, 10 μm (A). The bar graph shows the ratio of nuclear contouring in wild‐type and mutant lamin A‐expressing C2C12 cells. Cells were visualized 24 h after transfection, and the nuclear contouring ratio was quantified from photomicrographs using ImageJ (*n* = 10, each dot represents a mean value from a single fluorescence image slide acquired at x20 magnification, containing approximately 30–35 nuclei. *n* indicates the number of image slides analysed) ****p < 0.0001, ns: not significant by unpaired *t*‐test (B). His pull‐down assay using bead‐conjugated His‐tagged lamin A recombinant proteins (1–151 region) with C2C12 whole‐cell lysates expressing flag‐tagged LA‐WT, LA‐L35V, L38F and LA‐Y45C. The bar graph (right) indicates the relative binding affinity of EDMD‐related mutants to lamin A protein compared with wild‐type lamin A (*n* = 2, independent experiments) ****p < 0.0001 and **p < 0.01 by unpaired *t*‐test. Graph data were quantified using ‘Band Peak Quantification’ module in ImageJ software (C).
**Figure S5:** EDMD‐associated lamin A mutants possessing multilobular‐shaped nuclei are disconnected from BIN1. Immunoprecipitation (IP) assay using anti‐V5 antibody. RD whole lysates, cotransfected with V5‐tagged BIN1 and flag‐tagged lamin A variants, were incubated with anti‐V5 antibody for 2 h at 4°C. EV refers to empty vector, which is used as a negative control (A). His pull‐down assay using bead‐conjugated His‐tagged lamin A‐L35V, lamin A‐L38F, and lamin A‐Y45C recombinant proteins (1–151 region) with RD whole‐cell lysates expressing V5‐tagged BIN1 (B). IP assay using anti‐V5 antibody. RD whole lysates, cotransfected with V5‐tagged BIN1 and wild‐type lamin A or flag‐tagged lamin A mutants (L35V, L38F and Y45C), were incubated with anti‐V5 antibody for 2 h at 4°C (C). The bar graph shows the relative binding affinity of EDMD‐related lamin A mutants to BIN1 compared with wild‐type lamin A (*n* = 2, independent experiments) ****p < 0.0001 and **p < 0.01 by unpaired *t*‐test. Graph data were quantified using ‘Band Peak Quantification’ module in ImageJ software (D). GST pull‐down assay using bead‐conjugated GST‐tagged (skeletal muscle‐related) BIN1 recombinant proteins with C2C12 whole‐cell lysates expressing flag‐tagged wild‐type lamin A and EDMD‐related mutants. The bar graph (right) indicates the relative binding affinity of lamin A mutants to BIN1 (*n* = 2, independent experiments) ****p < 0.0001 and ***p < 0.001 by unpaired *t*‐test. Graph data were quantified using ‘Band Peak Quantification’ module in ImageJ software (E). Bar graph of relative cytoplasmic BIN1 expression in RD cells after transfection with wild‐type lamin A or EDMD‐related mutants (*n* = 2, independent experiments) *p < 0.05 by unpaired *t*‐test. Graph data were quantified using ‘Band Peak Quantification’ module in ImageJ software (F).
**Figure S6:** Disorganized CLIP1 in EDMD‐associated lamin A mutants. Immunofluorescence (IF) analysis of CLIP1 (green) expression in transiently cotransfected RD cells with mEmerald‐tagged CLIP1 and flag‐tagged wild‐type lamin A or mutants (L35V, L38F and Y45C). Cells were immunostained for Flag (red) and stained with DAPI (blue). Scale bar, 10 μm.
**Figure S7:** Disorganized BIN1 and *γ*‐tubulin in EDMD‐associated lamin A mutants. Immunofluorescence analysis of BIN1 (green) and *γ*‐tubulin (red) expression in transiently transfected RD cells with GFP‐tagged BIN1 and wild‐type lamin A or mutants (L35V, L38F and Y45C). Cells were immunostained for *γ*‐tubulin (red) and stained with DAPI (blue). Scale bar, 10 μm (A). The bar graph indicates the intensity of *γ*‐tubulin in RD cells (*n* = 10, each dot represents a mean value from a single fluorescence image slide acquired at x20 magnification, containing approximately 50–60 nuclei. *n* indicates the number of image slides analysed) **p < 0.01 and *p < 0.05, ns: not significant by unpaired *t*‐test. Graph data were quantified using ‘Band Peak Quantification’ module in ImageJ software (B).
**Figure S8:** Decrease of SUN1 expression in EDMD‐related lamin A mutants. The expression of SUN1 was decreased in EDMD‐related lamin A mutants. RD cells were transiently transfected with flag‐tagged wild‐type lamin A or mutant lamin A vectors for western blot analysis. Whole‐cell lysates from RD cells were subjected to SDS‐PAGE and immunoblotting with antibodies targeting flag, SUN1, BIN1 and Nesprin2 (A). The bar graph shows the relative SUN1 expression in RD cells (*n* = 2, independent experiments) *p < 0.05 and **p < 0.01 by unpaired *t*‐test. Graph data were quantified using ‘Band Peak Quantification’ module in ImageJ software (B)
**Figure S9:** Generation of MSCs derived from iPSCs using fibroblasts from a patient with EDMD. Immunofluorescence analysis of nuclear structure in fibroblasts from a healthy normal subject and an EDMD patient. Both fibroblasts were immunostained with antilamin A/C antibody and stained with DAPI (blue). Scale bar, 10 μm. The bar graph (right) shows the nuclear size (area) of fibroblasts from a healthy unaffected subject (healthy normal) and an EDMD patient (L35P). Nuclear size was quantified using ImageJ software (*n* = 10, each dot represents a mean value from a single fluorescence image slide acquired at x20 magnification, containing approximately 20–30 nuclei. *n* indicates the number of image slides analysed) *p < 0.05 by unpaired *t*‐test (A). Morphological changes during differentiation of human iPSCs into MSCs. Scale bar, 100 μm (B). Identification of iPSCs generated from EDMD fibroblasts by RT‐PCR. Induction of stem cell factors (KLF4, SOX2, OCT4, c‐Myc and Nanog) expression in EDMD iPSCs (C). Identification of MSCs differentiated from iPSCs. Reduction of stem cell factors and induction of Brachyury expression in EDMD‐MSCs (D).
**Figure S10:** MSCs derived from an EDMD patient show disrupted cytoskeletal structures. Immunofluorescence (IF) analysis of microtubules (A) and actin filaments (B) in healthy normal MSCs (Nor‐MSC) and EDMD patient‐derived MSCs (L35P‐MSC). Cells were immunostained for lamin A/C (green) and *α*‐tubulin (red) (A). The actin filaments were stained with phalloidin (red) (B). The nuclei were labelled with DAPI (blue). Scale bar, 10 μm.
**Figure S11:** MSCs derived from an EDMD patient display collapsed nuclear envelopes. Immunofluorescence (IF) analysis of emerin (A) and lamin B1 (B) expression in MSCs. Cells were immunostained with antiemerin (red) and antilamin A/C (green) antibodies in (A) and were immunostained with antilamin A/C (red) and antilamin B1 (green) antibodies in (B). The nuclei were labelled with DAPI (blue). Scale bar, 10 μm.
**Figure S12:** MSCs derived from an EDMD patient show altered protein expression and impaired binding affinity compared with MSCs derived from a healthy subject. Expression of lamin B1, SUN1, Nesprin2, CLIP1, BIN1 and H3K9ac was examined by western blot in Nor‐MSCs and L35P‐MSCs. The bar graph (right) indicates relative protein expression levels of SUN1, CLIP1 and H3K9ac in Nor‐MSCs and L35P‐MSCs (*n* = 3, independent experiments) ****p < 0.0001, **p < 0.01 and *p < 0.05 by unpaired *t*‐test. Graph data were quantified using ‘Band Peak Quantification’ module in ImageJ software (A). Immunoprecipitation (IP) assay using antilamin A/C antibody. Whole lysates of Nor‐MSCs and L35P‐MSCs were incubated with antilamin A/C antibody overnight at 4°C (B). The bar graphs indicate relative binding affinity of lamin A/C to SUN1 (left) and to BIN1 (right) in Nor‐MSCs and L35P‐MSCs (*n* = 2, independent experiments) ***p < 0.001 by unpaired *t*‐test. Graph data were quantified using ‘Band Peak Quantification’ module in ImageJ software (C)
**Figure S13:** MSCs derived from an EDMD patient show increased mechanosensitive response and nuclear fragility compared with MSCs derived from a healthy subject. Immunofluorescence (IF) analysis of YAP1 (red) expression in Nor‐MSCs and L35P‐MSCs. Cells were immunostained for lamin A/C (green) and YAP1 (red) after treatment with CoCl_2_ (10 μM) for 2 h (A). Analysis of nuclear fragility by immunofluorescence assay in Nor‐MSCs and L35P‐MSCs after physical stimulation (pressure) for 24 h. Cells were immunostained with antilamin A/C (green) after the physical stimulation assay. White arrowheads indicate regions of nuclear blebbing or nuclear rupture (B). The nuclei were labelled with DAPI (blue). Scale bar, 10 μm.
**Figure S14:** ASOs restore nuclear abnormalities in EDMD‐MSCs. The bar graph shows the relative cell viability after long‐term overexpression (5 days) in RD cells. Transfection was performed twice over a period of five days. ASO treatment was performed twice over the 5‐day period, with each administration given 24 h after each round of vector transfection. Cell viability was measured by MTT assay (*n* = 2, independent experiments) ***p < 0.001 by unpaired *t*‐test (A). Immunofluorescence (IF) analysis for nuclear morphology after being treated with ASOs in L35P‐MSCs were immunostained with antilamin A/C (red) and antilamin B1 (green) after transfection with NC‐ASOs and L35P‐targeting ASOs for 48 h (B). The bar graphs indicate relative protein expression levels of SUN1 (upper) and H3K9ac (lower) in Nor‐MSCs and L35P‐MSCs (*n* = 2, independent experiments) *p < 0.05 by unpaired *t*‐test. Graph data were quantified using ‘Band Peak Quantification’ module in ImageJ software (C). Immunoprecipitation (IP) assay using antilamin A/C antibody. Whole lysates of Nor‐MSCs and L35P‐MSCs after ASO treatment were incubated with antilamin A/C antibody overnight at 4°C (D). The bar graphs indicate relative binding affinity of lamin A/C to SUN1 (left), to Nesprin2 (middle) and to BIN1 (right) in Nor‐MSCs and L35P‐MSCs (*n* = 2, independent experiments) **p < 0.01 and *p < 0.05 by unpaired *t*‐test. Graph data were quantified using ‘Band Peak Quantification’ module in ImageJ software (E).
**Figure S15:** ASOs reestablish cytoskeletal structure in EDMD‐MSCs. Immunofluorescence (IF) analysis of microtubules (A) and actin filaments (B) in L35P‐MSCs after ASO treatment. Cells were fixed and immunostained for lamin A/C (green) and *α*‐tubulin (red) (A). The actin filaments were stained with phalloidin (red) (B). The nuclei were labelled with DAPI (blue). Scale bar, 10 μm.
**Figure S16:** ASOs restore the mechanosensitive response in EDMD‐MSCs. Immunofluorescence (IF) analysis of YAP1 (red) expression in Nor‐MSCs and L35P‐MSCs after ASO treatment. After 48 h of ASO treatment, cells were treated with CoCl_2_ (10 μM) for 2 h before fixation and subsequently immunostained for lamin A/C (green) and YAP1 (red). The nuclei were labelled with DAPI (blue). Scale bar, 10 μm (A). The bar graph indicates the intensity of nuclear YAP1 in Nor‐MSCs and L35P‐MSCs after ASO treatment (*n* = 5, each dot represents a mean value from a single fluorescence image slide acquired at x20 magnification, containing approximately 20–30 nuclei. *n* indicates the number of image slides analysed) **p < 0.01 by unpaired *t*‐test (B).
**Figure S17:** ASOs reduce nuclear fragility in EDMD‐MSCs. Immunofluorescence (IF) analysis of nuclear fragility in Nor‐MSCs and L35P‐MSCs following ASO treatment and exposure to physical (pressure) stimulation. After 48 h of ASO treatment, MSCs were subjected to physical stimulation for 24 h and subsequently immunostained with antilamin A/C (green) antibody. The nuclei were labelled with DAPI (blue). Scale bar, 10 μm (A). The graph shows the percentage of nuclear fragility, with or without pressure, in Nor‐MSCs and L35P‐MSCs after ASO treatment. Nuclear fragility was quantified by counting nuclei exhibiting nuclear blebbing or rupture under physical stimulation (*n* = 10, each dot represents a mean value from a single fluorescence image slide acquired at x20 magnification, containing approximately 20–30 nuclei. *n* indicates the number of image slides analysed) ****p < 0.0001 by unpaired *t*‐test (B).
**Figure S18:** Comparison of nuclear disruption in other lamin A mutations related to EDMD. Immunofluorescence analysis for nuclear morphology after transfection with wild‐type lamin A (LA‐WT) and EDMD‐related lamin A variants (H222Y, R386K and L530P). GFP‐tagged wild‐type lamin A and lamin A mutants (R386K and L530P) expressing RD cells were immunostained with antilamin A/C antibody. Flag‐tagged wild‐type lamin A and lamin A mutant (H222Y) expressing RD cells were immunostained with antiflag and antilamin A/C antibodies. Nuclei were stained with DAPI (blue). The bar graph (right) shows the ratio of nuclear contouring lamin A mutants (LA‐H222Y, LA‐R386K and LA‐L530P) expressing cells compared with wild‐type lamin A expressing cells (*n* = 12, each dot represents a mean value from a single fluorescence image slide acquired at x20 magnification, containing approximately 40–50 nuclei. *n* indicates the number of image slides analysed) ****p < 0.0001, ns: not significant by unpaired *t*‐test. Scale bar, 10 μm (A). His pull‐down assay using RD whole‐cell lysates expressing flag‐tagged LA‐WT and LA‐H222Y, or GFP‐tagged LA‐WT, LA‐R386K and LA‐L530P. Each lysate was incubated with bead‐conjugated His‐tagged lamin A (1–151 region) recombinant proteins (B). The bar graph shows the relative binding affinity of EDMD‐related mutants to lamin A protein (*n* = 2, independent experiments) ****p < 0.0001 and *p < 0.05 by unpaired *t*‐test. Graph data were quantified using ‘Band Peak Quantification’ module in ImageJ software (C).
