# Supplementary material for: Disruption of Nuclear‐Cytoskeletal Linkage by Coil‐1a LMNA Mutations in Emery–Dreifuss Muscular Dystrophy
Source: J Cachexia Sarcopenia Muscle. 2026 Feb 17;17(1):e70234. doi: 10.1002/jcsm.70234 (PMC12914145; doi:10.1002/jcsm.70234)
Supplement: Supplementary file 1 — Data S1: Supporting information. [file JCSM-17-e70234-s003.docx]

**Supplementary Information**

**Disruption of nuclear-cytoskeletal linkage by Coil-1a LMNA mutations in Emery-Dreifuss Muscular Dystrophy**

So-mi Kang^1,*^, Ran Kim^2^, Tae-Gyun Woo^3^, So-Young Park^3^, Yeongseon Ji^1^, Ha Eun Kim^1^, Yu Jin Jeong^1^, Jeongmo Kim^1^, Yeonhee Kim^1^, Woochul Chang^2^, Bae-Hoon Kim^3^, Bum-Joon Park^1,3,#^

**Affiliations**

1. Department of Molecular Biology, Pusan National University, Busan, Korea (Republic of)
2. Department of Biology Education, Pusan National University, Busan, Korea (Republic of)
3. Rare Disease R&D Center, PRG S&T Co., Ltd., Busan, Korea (Republic of)

# Correspondence: [bipark1219@pusan.ac.kr](mailto:bipark1219@pusan.ac.kr) (Bum-Joon Park)

**Additional references**

1. **Worman, H. J., Fong, L. G., Muchir, A. & Young, S. G. Laminopathies and the long strange trip from basic cell biology to therapy. *J. Clin. Invest.* 119, 1825–1836 (2009).**
2. **Vigouroux, C. & Bonne, G. in *Madame Curie Bioscience Database [Internet]* (Landes Bioscience, 2013).**
3. **Helbling-Leclerc, A., Bonne, G. & Schwartz, K. Emery-Dreifuss muscular dystrophy. *European Journal of Human Genetics* 10, 157–161 (2002).**
4. **Iskandar, K. *et al*. Autosomal dominant Emery-Dreifuss muscular dystrophy caused by a mutation in the lamin A/C gene identified by exome sequencing: a case report. *BMC pediatrics* 22, 601 (2022).**
5. **Zhao, T., Graham, O. S., Raposo, A. & St Johnston, D. Growing microtubules push the oocyte nucleus to polarize the Drosophila dorsal-ventral axis. *Science* 336, 999–1003 (2012).**
6. **Gueneau, L. *et al*. Mutations of the FHL1 gene cause Emery-Dreifuss muscular dystrophy. *The American Journal of Human Genetics* 85, 338–353 (2009).**
7. **Aartsma-Rus, A. *et al*. Consensus guidelines for the design and in vitro preclinical efficacy testing N-of-1 exon skipping antisense oligonucleotides. Nucleic Acid Ther 33: 17–25. *FDA nonclinical* (2022).**
8. **Zheng, M., Jin, G. & Zhou, Z. Post-translational modification of lamins: mechanisms and functions. *Frontiers in Cell and Developmental Biology* 10, 864191 (2022).**
9. **Liu, S. Y. & Ikegami, K. Nuclear lamin phosphorylation: an emerging role in gene regulation and pathogenesis of laminopathies. *Nucleus* 11, 299–314 (2020).**
10. **Arsenovic, P. T. *et al*. Nesprin-2G, a component of the nuclear LINC complex, is subject to myosin-dependent tension. *Biophys. J.* 110, 34–43 (2016).**
11. **Li, P. *et al*. The function of the inner nuclear envelope protein SUN1 in mRNA export is regulated by phosphorylation. *Scientific reports* 7, 9157 (2017).**
12. **Puckelwartz, M. J., Depreux, F. F. & McNally, E. M. Gene expression, chromosome position and lamin A/C mutations. *Nucleus* 2, 14342–14329 (2011).**
13. **Lammerding, J. *et al*. Lamins A and C but not lamin B1 regulate nuclear mechanics. *J. Biol. Chem.* 281, 25768–25780 (2006).**
14. **Kalukula, Y., Stephens, A. D., Lammerding, J. & Gabriele, S. Mechanics and functional consequences of nuclear deformations. *Nature reviews Molecular cell biology* 23, 583–602 (2022).**
15. **Veltrop, R. *et al*. From gene to mechanics: a comprehensive insight into the mechanobiology of LMNA mutations in cardiomyopathy. *Cell Communication and Signaling* 22, 197 (2024).**
16. **Wallace, M. *et al*. Nuclear damage in LMNA mutant iPSC-derived cardiomyocytes is associated with impaired lamin localization to the nuclear envelope. *Mol. Biol. Cell* 34, ar113 (2023).**
17. **Heller, S. A., Shih, R., Kalra, R. & Kang, P. B. Emery‐Dreifuss muscular dystrophy. *Muscle Nerve* 61, 436–448 (2020).**
18. **Muchir, A. & Worman, H. J. Emery–Dreifuss muscular dystrophy: focal point nuclear envelope. *Curr. Opin. Neurol.* 32, 728–734 (2019).**
19. **DuBose, A. J. *et al*. Everolimus rescues multiple cellular defects in laminopathy-patient fibroblasts. *Proceedings of the National Academy of Sciences* 115, 4206–4211 (2018).**
20. **Vytopil, M. *et al*. Mutation analysis of the lamin A/C gene (LMNA) among patients with different cardiomuscular phenotypes. *J. Med. Genet.* 40, e132 (2003).**
21. **Zammit, P. *et al*. Novel< i> LMNA mutations in patients with Emery-Dreifuss muscular dystrophy and functional characterization of four< i> LMNA mutations. (2011).**
22. **di Barletta, M. R. *et al*. Different mutations in the LMNA gene cause autosomal dominant and autosomal recessive Emery-Dreifuss muscular dystrophy. *The American Journal of Human Genetics* 66, 1407–1412 (2000).**
23. **Wu, T., Shi, Z. & Baumgart, T. Mutations in BIN1 associated with centronuclear myopathy disrupt membrane remodeling by affecting protein density and oligomerization. *PloS one* 9, e93060 (2014).**
24. **Ali, A., Vineethakumari, C., Lacasa, C. & Lüders, J. Microtubule nucleation and γTuRC centrosome localization in interphase cells require ch-TOG. *Nature Communications* 14, 289 (2023).**
25. **Yagoubat, A. & Conduit, P. T. Asymmetric microtubule nucleation from Golgi stacks promotes opposite microtubule polarity in axons and dendrites. *Current Biology* 35, 1311–1325. e4 (2025).**
26. **Yagoubat, A. & Conduit, P. T. Asymmetric microtubule nucleation from Golgi stacks promotes opposite microtubule polarity in axons and dendrites. *Current Biology* 35, 1311–1325. e4 (2025).**
