# Supplementary material for: Disruption of Nuclear‐Cytoskeletal Linkage by Coil‐1a LMNA Mutations in Emery–Dreifuss Muscular Dystrophy
Source: J Cachexia Sarcopenia Muscle. 2026 Feb 17;17(1):e70234. doi: 10.1002/jcsm.70234 (PMC12914145; doi:10.1002/jcsm.70234)
Supplement: Supplementary file 3 — Figure S1: Expression of EDMD‐associated lamin A mutants results in nuclei with a characteristic multilobulated morphology. RD cells expressing wild‐type (LA‐WT) and EDMD‐related lamin A mutants (LA‐L35V, LA‐L38F and Y45C) were immunostained for flag (green) and lamin A/C (red), and stained with DAPI (blue). Cells were visualized at 24 h after transfection. Scale bar, 10 μm (A). Schematic illustration showing the relationship between nuclear morphology and nuclear contour ratio. A value closer to 1 represents a rounded nucleus, whereas a lower ratio indicates a deformed nuclear shape (B). Figure S2: EDMD‐associated lamin A mutants induce different spatial distributions. The bar graph indicates the relative expression of phospholamin A/C (S22) of wild‐type lamin A and EDMD‐related mutants in RD cells (n = 2, independent experiments) **p < 0.01 and *p < 0.05, ns: not significant by unpaired t‐test. Graph data were quantified using ‘Band Peak Quantification’ module in ImageJ software (A). Western blot analysis of nuclear and cytoplasmic fractions using NP‐40 lysis buffer. PEL, nuclear fraction; SUP, cytoplasmic fraction. Samples were centrifuged at 3000 rpm and the pellet (PEL) and supernatant (SUP) were collected separately (B). The stacked bar graph represents relative proportions of nuclear (dark purple) and cytosolic (light purple) fractions in wild‐type lamin A and EDMD‐related mutants. The stacked bar graph presents the quantification of relative Flag expression in the ‘PEL’ and ‘SUP’ lanes based on the western blot results shown in Figure S1D (C). The bar graph shows the relative cell viability after long‐term overexpression (5 days) in RD cells. Transfection was performed twice over a period of five days. Cell viability was measured by MTT assay (n = 2, independent experiments) ****p < 0.0001 and **p < 0.01 by unpaired t‐test (D). The bar graph shows the relative binding affinity of EDMD‐related mutants to lamin A protein compared with wild‐type lamin A (n = 2, [file JCSM-17-e70234-s002.docx]

**Supplementary Figure 1. Expression of EDMD-associated lamin A mutants results in nuclei with a characteristic multilobulated morphology.** RD cells expressing wild-type (LA-WT) and EDMD-related lamin A mutants (LA-L35V, LA-L38F, and Y45C) were immunostained for Flag (green) and lamin A/C (red), and stained with DAPI (blue). Cells were visualized at 24 h after transfection. Scale bar, 10 µm (A). Schematic illustration showing the relationship between nuclear morphology and nuclear contour ratio. A value closer to 1 represents a rounded nucleus, whereas a lower ratio indicates a deformed nuclear shape (B).

**Supplementary Figure 2. EDMD-associated lamin A mutants induce different spatial distributions.** The bar graph indicates the relative expression of phospho-lamin A/C (S22) of wild-type lamin A and EDMD-related mutants in RD cells (n=2, independent experiments) ***p*<0.01, **p*<0.05, ns: not significant by unpaired *t*-test. Graph data were quantified using ‘Band Peak Quantification’ module in ImageJ software (A). Western blot analysis of nuclear and cytoplasmic fractions using NP-40 lysis buffer. PEL, nuclear fraction; SUP, cytoplasmic fraction. Samples were centrifuged at 3,000 rpm, and the pellet (PEL) and supernatant (SUP) were collected separately (B). The stacked bar graph represents relative proportions of nuclear (dark purple) and cytosolic (light purple) fractions in wild-type lamin A and EDMD-related mutants. The stacked bar graph presents the quantification of relative Flag expression in the ‘PEL’ and ‘SUP’ lanes based on the Western blot results shown in Supplementary Figure 1D (C). The bar graph shows the relative cell viability after long-term overexpression (5 days) in RD cells. Transfection was performed twice over a period of five days. Cell viability was measured by MTT assay (n=2, independent experiments) *****p*<0.0001, ***p*<0.01 by unpaired *t*-test (D). The bar graph shows the relative binding affinity of EDMD-related mutants to lamin A protein compared with wild-type lamin A (n=2, independent experiments) ***p*<0.01, **p*<0.05 by unpaired *t*-test. Graph data were quantified using ‘Band Peak Quantification’ module in ImageJ software (E).

**Supplementary Figure 3. EDMD-associated lamin A mutants induce binding abnormalities.** His pull-down assay using bead-conjugated His-tagged lamin A-L35V (A), lamin A-L38F (B), or lamin A-Y45C (C) recombinant proteins (1-151 region) with RD whole cell lysates expressing Flag-tagged LA-WT, LA-L35V, L38F, and LA-Y45C. Each bar graph (right) indicates the relative binding affinity to each lamin A-mutant protein (n=2, independent experiments) ****p*<0.001, ***p*<0.01, **p*<0.05 by unpaired *t*-test. Graph data were quantified using ‘Band Peak Quantification’ module in ImageJ software.

Supplementary Figure 4. EDMD-associated lamin A mutants induce nuclear abnormalities and binding deficits in mouse myoblasts. C2C12 cells expressing Flag-tagged wild-type lamin A (LA-WT) and EDMD-related mutants (LA-L35V, LA-L38F, and LA-Y45C) were fixed and immunostained for Flag (green), Lamin A/C (red), and stained with DAPI (blue). Scale bar, 10 µm (A). The bar graph shows the ratio of nuclear contouring in wild-type and mutant lamin A-expressing C2C12 cells. Cells were visualized 24 h after transfection, and the nuclear contouring ratio was quantified from photomicrographs using ImageJ (n=10, each dot represents a mean value from a single fluorescence image slide acquired at x20 magnification, containing approximately 30-35 nuclei. ‘n’ indicates the number of image slides analyzed) *****p*<0.0001, ns: not significant by unpaired *t*-test (B). His pull-down assay using bead-conjugated His-tagged lamin A recombinant proteins (1-151 region) with C2C12 whole cell lysates expressing Flag-tagged LA-WT, LA-L35V, L38F, and LA-Y45C. The bar graph (right) indicates the relative binding affinity of EDMD-related mutants to lamin A protein compared with wild-type lamin A (n=2, independent experiments) *****p*<0.0001, ***p*<0.01 by unpaired *t*-test. Graph data were quantified using ‘Band Peak Quantification’ module in ImageJ software (C).

**Supplementary Figure 5. EDMD-associated lamin A mutants possessing multi-lobular-shaped nuclei are disconnected from BIN1.** Immunoprecipitation (IP) assay using anti-V5 antibody. RD whole lysates, co-transfected with V5-tagged BIN1 and Flag-tagged lamin A variants, were incubated with anti-V5 antibody for 2 h at 4℃. EV refers to empty vector, which is used as a negative control (A). His pull-down assay using bead-conjugated His-tagged lamin A-L35V, lamin A-L38F, and lamin A-Y45C recombinant proteins (1-151 region) with RD whole cell lysates expressing V5-tagged BIN1 (B). IP assay using anti-V5 antibody. RD whole lysates, co-transfected with V5-tagged BIN1 and wild-type lamin A or Flag-tagged lamin A mutants (L35V, L38F, and Y45C), were incubated with anti-V5 antibody for 2 h at 4℃ (C). The bar graph shows the relative binding affinity of EDMD-related lamin A mutants to BIN1 compared with wild-type lamin A (n=2, independent experiments) *****p*<0.0001, ***p*<0.01 by unpaired *t*-test. Graph data were quantified using ‘Band Peak Quantification’ module in ImageJ software (D). GST pull-down assay using bead-conjugated GST-tagged (skeletal muscle-related) BIN1 recombinant proteins with C2C12 whole cell lysates expressing Flag-tagged wild-type lamin A and EDMD-related mutants. The bar graph (right) indicates the relative binding affinity of lamin A mutants to BIN1 (n=2, independent experiments) *****p*<0.0001, ****p*<0.001 by unpaired *t*-test. Graph data were quantified using ‘Band Peak Quantification’ module in ImageJ software (E). Bar graph of relative cytoplasmic BIN1 expression in RD cells after transfection with wild-type lamin A or EDMD-related mutants (n=2, independent experiments) **p*<0.05 by unpaired *t*-test. Graph data were quantified using ‘Band Peak Quantification’ module in ImageJ software (F).

**Supplementary Figure 6. Disorganized CLIP1 in EDMD-associated lamin A mutants.** Immunofluorescence (IF) analysis of CLIP1 (green) expression in transiently co-transfected RD cells with mEmerald-tagged CLIP1 and Flag-tagged wild-type lamin A or mutants (L35V, L38F, and Y45C). Cells were immunostained for Flag (red) and stained with DAPI (blue). Scale bar, 10 µm.

**Supplementary Figure 7. Disorganized BIN1 and γ-tubulin in EDMD-associated lamin A mutants.** Immunofluorescence analysis of BIN1 (green) and γ-tubulin (red) expression in transiently transfected RD cells with GFP-tagged BIN1 and wild-type lamin A or mutants (L35V, L38F, and Y45C). Cells were immunostained for γ-tubulin (red) and stained with DAPI (blue). Scale bar, 10 µm (A). The bar graph indicates the intensity of γ-tubulin in RD cells (n=10, each dot represents a mean value from a single fluorescence image slide acquired at x20 magnification, containing approximately 50-60 nuclei. ‘n’ indicates the number of image slides analyzed) ***p*<0.01, **p*<0.05, ns: not significant by unpaired *t*-test. Graph data were quantified using ‘Band Peak Quantification’ module in ImageJ software (B).

**Supplementary Figure 8. Decrease of SUN1 expression in EDMD-related lamin A mutants.** The expression of SUN1 was decreased in EDMD-related lamin A mutants. RD cells were transiently transfected with Flag-tagged wild-type lamin A or mutant lamin A vectors for western blot analysis. Whole-cell lysates from RD cells were subjected to SDS-PAGE and immunoblotting with antibodies targeting Flag, SUN1, BIN1, and Nesprin2 (A). The bar graph shows the relative SUN1 expression in RD cells (n=2, independent experiments) **p*<0.05, ***p*<0.01 by unpaired *t*-test. Graph data were quantified using ‘Band Peak Quantification’ module in ImageJ software (B).

**Supplementary Figure 9. Generation of MSCs derived from iPSCs using fibroblasts from a patient with EDMD.** Immunofluorescence analysis of nuclear structure in fibroblasts from a healthy normal subject and an EDMD patient. Both fibroblasts were immunostained with anti-lamin A/C antibody and stained with DAPI (blue). Scale bar, 10 µm. The bar graph (right) shows the nuclear size (area) of fibroblasts from a healthy unaffected subject (healthy normal) and an EDMD patient (L35P). Nuclear size was quantified using ImageJ software (n=10, each dot represents a mean value from a single fluorescence image slide acquired at x20 magnification, containing approximately 20-30 nuclei. ‘n’ indicates the number of image slides analyzed) **p*<0.05 by unpaired *t*-test (A). Morphological changes during differentiation of human iPSCs into MSCs. Scale bar, 100 µm (B). Identification of iPSCs generated from EDMD fibroblasts by RT-PCR. Induction of stem cell factors (KLF4, SOX2, OCT4, c-Myc, and Nanog) expression in EDMD iPSCs (C). Identification of MSCs differentiated from iPSCs. Reduction of stem cell factors and induction of Brachyury expression in EDMD MSCs (D).

**Supplementary Figure 10. MSCs derived from an EDMD patient show disrupted cytoskeletal structures.** Immunofluorescence (IF) analysis of microtubules (A) and actin filaments (B) in healthy normal MSCs (Nor-MSC) and EDMD patient-derived MSCs (L35P-MSC). Cells were immunostained for lamin A/C (green) and α-tubulin (red) (A). The actin filaments were stained with phalloidin (red) (B). The nuclei were labeled with DAPI (blue). Scale bar, 10 µm.

**Supplementary Figure 11. MSCs derived from an EDMD patient display collapsed nuclear envelopes.** Immunofluorescence (IF) analysis of emerin (A) and lamin B1 (B) expression in MSCs. Cells were immunostained with anti-emerin (red) and anti-lamin A/C (green) antibodies in (A) and were immunostained with anti-lamin A/C (red) and anti-lamin B1 (green) antibodies in (B). The nuclei were labeled with DAPI (blue). Scale bar, 10 µm.

**Supplementary Figure 12. MSCs derived from an EDMD patient show altered protein expression and impaired binding affinity compared with MSCs derived from a healthy subject.** Expression of lamin B1, SUN1, Nesprin2, CLIP1, BIN1, and H3K9ac was examined by western blot in Nor-MSCs and L35P-MSCs. The bar graph (right) indicates relative protein expression levels of SUN1, CLIP1, and H3K9ac in Nor-MSCs and L35P-MSCs (n=3, independent experiments) *****p*<0.0001, ***p*<0.01, **p*<0.05 by unpaired *t*-test. Graph data were quantified using ‘Band Peak Quantification’ module in ImageJ software (A). Immunoprecipitation (IP) assay using anti-lamin A/C antibody. Whole lysates of Nor-MSCs and L35P-MSCs were incubated with anti-lamin A/C antibody overnight at 4℃ (B). The bar graphs indicate relative binding affinity of lamin A/C to SUN1 (left) and to BIN1 (right) in Nor-MSCs and L35P-MSCs (n=2, independent experiments) ****p*<0.001 by unpaired *t*-test. Graph data were quantified using ‘Band Peak Quantification’ module in ImageJ software (C).

**Supplementary Figure 13. MSCs derived from an EDMD patient show increased mechanosensitive response and nuclear fragility compared with MSCs derived from a healthy subject.** Immunofluorescence (IF) analysis of YAP1 (red) expression in Nor-MSCs and L35P-MSCs. Cells were immunostained for lamin A/C (green) and YAP1 (red) after treatment with CoCl_2_ (10µM) for 2 h (A). Analysis of nuclear fragility by immunofluorescence assay in Nor-MSCs and L35P-MSCs after physical stimulation (pressure) for 24 h. Cells were immunostained with anti-lamin A/C (green) after the physical stimulation assay. White arrowheads indicate regions of nuclear blebbing or nuclear rupture (B). The nuclei were labeled with DAPI (blue). Scale bar, 10 µm.

**Supplementary Figure 14. ASOs restore nuclear abnormalities in EDMD-MSCs.** The bar graph shows the relative cell viability after long-term overexpression (5 days) in RD cells. Transfection was performed twice over a period of five days. ASO treatment was performed twice over the 5-day period, with each administration given 24 h after each round of vector transfection. Cell viability was measured by MTT assay (n=2, independent experiments) ****p*<0.001 by unpaired *t*-test (A). Immunofluorescence (IF) analysis for nuclear morphology after being treated with ASOs in L35P-MSCs were immunostained with anti-lamin A/C (red) and anti-lamin B1 (green) after transfection with NC-ASOs and L35P-targeting ASOs for 48 h (B). The bar graphs indicate relative protein expression levels of SUN1 (upper) and H3K9ac (lower) in Nor-MSCs and L35P-MSCs (n=2, independent experiments) **p*<0.05 by unpaired *t*-test. Graph data were quantified using ‘Band Peak Quantification’ module in ImageJ software (C). Immunoprecipitation (IP) assay using anti-lamin A/C antibody. Whole lysates of Nor-MSCs and L35P-MSCs after ASO treatment were incubated with anti-lamin A/C antibody overnight at 4℃ (D). The bar graphs indicate relative binding affinity of lamin A/C to SUN1 (left), to Nesprin2 (middle), and to BIN1 (right) in Nor-MSCs and L35P-MSCs (n=2, independent experiments) ***p*<0.01, **p*<0.05 by unpaired *t*-test. Graph data were quantified using ‘Band Peak Quantification’ module in ImageJ software (E).

**Supplementary Figure 15. ASOs reestablish cytoskeletal structure in EDMD-MSCs.** Immunofluorescence (IF) analysis of microtubules (A) and actin filaments (B) in L35P-MSCs after ASO treatment. Cells were fixed and immunostained for lamin A/C (green) and α-tubulin (red) (A). The actin filaments were stained with phalloidin (red) (B). The nuclei were labeled with DAPI (blue). Scale bar, 10 µm.

**Supplementary Figure 16. ASOs restore the mechanosensitive response in EDMD-MSCs.** Immunofluorescence (IF) analysis of YAP1 (red) expression in Nor-MSCs and L35P-MSCs after ASO treatment. After 48 h of ASO treatment, cells were treated with CoCl_2_ (10µM) for 2 h before fixation and subsequently immunostained for lamin A/C (green) and YAP1 (red). The nuclei were labeled with DAPI (blue). Scale bar, 10 µm (A). The bar graph indicates the intensity of nuclear YAP1 in Nor-MSCs and L35P-MSCs after ASO treatment (n=5, each dot represents a mean value from a single fluorescence image slide acquired at x20 magnification, containing approximately 20-30 nuclei. ‘n’ indicates the number of image slides analyzed) ***p*<0.01 by unpaired *t*-test (B).

**Supplementary Figure 17. ASOs reduce nuclear fragility in EDMD-MSCs.** Immunofluorescence (IF) analysis of nuclear fragility in Nor-MSCs and L35P-MSCs following ASO treatment and exposure to physical (pressure) stimulation. After 48 h of ASO treatment, MSCs were subjected to physical stimulation for 24 h and subsequently immunostained with anti-lamin A/C (green) antibody. The nuclei were labeled with DAPI (blue). Scale bar, 10 µm (A). The graph shows the percentage of nuclear fragility, with or without pressure, in Nor-MSCs and L35P-MSCs after ASO treatment. Nuclear fragility was quantified by counting nuclei exhibiting nuclear blebbing or rupture under physical stimulation (n=10, each dot represents a mean value from a single fluorescence image slide acquired at x20 magnification, containing approximately 20-30 nuclei. ‘n’ indicates the number of image slides analyzed) *****p*<0.0001 by unpaired *t*-test. (B).

**Supplementary Figure 18. Comparison of nuclear disruption in other lamin A mutations related to EDMD.** Immunofluorescence analysis for nuclear morphology after transfection with wild-type lamin A (LA-WT) and EDMD-related lamin A variants (H222Y, R386K, and L530P). GFP-tagged wild-type lamin A and lamin A mutants (R386K and L530P) expressing RD cells were immunostained with anti-lamin A/C antibody. Flag-tagged wild-type lamin A and lamin A mutant (H222Y) expressing RD cells were immunostained with anti-Flag and anti-lamin A/C antibodies. Nuclei were stained with DAPI (blue). The bar graph (right) shows the ratio of nuclear contouring lamin A mutants (LA-H222Y, LA-R386K, and LA-L530P) expressing cells compared with wild-type lamin A expressing cells (n=12, each dot represents a mean value from a single fluorescence image slide acquired at x20 magnification, containing approximately 40-50 nuclei. ‘n’ indicates the number of image slides analyzed) *****p*<0.0001, ns: not significant by unpaired *t*-test. Scale bar, 10 µm (A). His pull-down assay using RD whole cell lysates expressing Flag-tagged LA-WT and LA-H222Y, or GFP-tagged LA-WT, LA-R386K, and LA-L530P. Each lysate was incubated with bead-conjugated His-tagged lamin A (1-151 region) recombinant proteins (B). The bar graph shows the relative binding affinity of EDMD-related mutants to lamin A protein (n=2, independent experiments) *****p*<0.0001, **p*<0.05 by unpaired *t*-test. Graph data were quantified using ‘Band Peak Quantification’ module in ImageJ software (C).
